# Preclinical pharmacokinetic characterization of amdizalisib, a novel PI3Kδ inhibitor for the treatment of hematological malignancies

**DOI:** 10.3389/fphar.2024.1392209

**Published:** 2024-06-14

**Authors:** Shuwen Jiang, Xiangkun Li, Weifang Xue, Sumei Xia, Jian Wang, Yang Sai, Guangxiu Dai, Weiguo Su

**Affiliations:** HUTCHMED Limited, Zhangjiang Hi-Tech Park, Shanghai, China

**Keywords:** PI3Kδ, hematological malignancies, pharmacokinetics, tissue distribution, *in vitro in vivo* extrapolation, drug–drug interaction

## Abstract

Amdizalisib, also named HMPL-689, a novel selective and potent PI3Kδ inhibitor, is currently under Phase II clinical development in China for treating hematological malignancies. The preclinical pharmacokinetics (PK) of amdizalisib were extensively characterized *in vitro* and *in vivo* to support the further development of amdizalisib. We characterized the plasma protein binding, blood-to-plasma partition ratio, cell permeability, hepatic microsomal metabolic stability, and drug–drug interaction potential of amdizalisib using *in vitro* experiments. *In vivo* PK assessment was undertaken in mice, rats, dogs, and monkeys following a single intravenous or oral administration of amdizalisib. The tissue distribution and excretion of amdizalisib were evaluated in rats. The PK parameters (CL and V_ss_) of amdizalisib in preclinical species (mice, rats, dogs, and monkeys) were utilized for the human PK projection using the allometric scaling (AS) approach. Amdizalisib was well absorbed and showed low-to-moderate clearance in mice, rats, dogs, and monkeys. It had high cell permeability without P-glycoprotein (P-gp) or breast cancer resistance protein (BCRP) substrate liability. Plasma protein binding of amdizalisib was high (approximately 90%). It was extensively distributed but with a low brain-to-plasma exposure ratio in rats. Amdizalisib was extensively metabolized *in vivo*, and the recovery rate of the prototype drug was low in the excreta. Amdizalisib and/or its metabolites were primarily excreted via the bile and urine in rats. Amdizalisib showed inhibition potential on P-gp but not on BCRP and was observed to inhibit CYP2C8 and CYP2C9 with IC_50_ values of 30.4 and 10.7 μM, respectively. It exhibited induction potential on CYP1A2, CYP2B6, CYP3A4, and CYP2C9. The preclinical data from these ADME studies demonstrate a favorable pharmacokinetic profile for amdizalisib, which is expected to support the future clinical development of amdizalisib as a promising anti-cancer agent.

## 1 Introduction

The phosphatidylinositol 3-kinases (PI3Ks) are members of intracellular lipid kinases that phosphorylate the 3′-hydroxyl (3′-OH) position of phosphoinositide lipids, leading to the activation of multiple intracellular signaling pathways that regulate cell proliferation, differentiation, apoptosis, glucose metabolism, and vesicle trafficking (Katso et al., 2001; Vivanco and Sawyers, 2002; Han et al., 2012; Bilanges et al., 2019). Over the past decade, most research findings have shown that aberrant activation of PI3K signaling pathways is one of the most common events in human cancers, underscoring its essential role in human carcinogenesis (Singh et al., 2015; Noorolyai et al., 2019; Miricescu et al., 2020). Substantial evidence indicates that PI3Ks play an important role in the initiation and progression of tumor progression, particularly in the control of cell proliferation, survival, and regulation of the potential oncogene protein kinase B (PKB) (Fayard et al., 2011; Koveitypour et al., 2019; Xue et al., 2021).

PI3Ks are divided into three main classes (Class I, II, and III) based on their substrate specificities and primary structural characteristics, with Class I being the most implicated in human cancers (Mishra et al., 2021). Class I contains four isoforms of the catalytic subunit, namely, p110α (PI3Kα), p110β (PI3Kβ), p110δ (PI3Kδ), and p110γ (PI3Kγ). The former two isoforms are ubiquitously expressed, while the expressions of the latter two isoforms are largely restricted to leukocytes (Beer-Hammer et al., 2010; Thorpe et al., 2015). B-cell receptor (BCR) signaling is a fundamental regulator of tumor survival in several B-cell lymphomas, including diffuse large B-cell lymphoma (DLBCL), chronic lymphocytic leukemia/small lymphocytic lymphoma (CLL/SLL), follicular lymphoma (FL), mantle cell lymphoma (MCL), and marginal zone lymphoma (MZL) (Burger and Wiestner, 2018). It is reported that the PI3Kδ isoform can be activated by the BCR transducing survival and proliferation signals via the nuclear factor kappa B (NF-κB) pathway, which plays a significant role in the development and clonal expansion of normal and malignant B-cells (Okkenhaug and Vanhaesebroeck, 2003; Seda and Mraz, 2015; Wiestner, 2015). Therefore, PI3Kδ is an effective target in the treatment of hematological malignancies.

In the past decades, molecularly targeted therapies, including PI3K inhibitors (PI3Kis), have changed the treatment landscape of lymphoma (Sapon-Cousineau et al., 2020; Berning and Lenz, 2021). Many pharmaceutical companies and research institutions are making efforts to develop potent and selective PI3Kδ inhibitors to treat B-cell malignancies. Idelalisib/Zydelig, an oral PI3Kδ inhibitor, was the world’s first selective PI3Kδ inhibitor approved by the US FDA in 2014 for the treatment of FL, CLL, and SLL (Yang et al., 2015). Data from phase I and II trials of idelalisib have indicated that early and late gastrointestinal (GI) toxicity, specifically diarrhea, is common and can be severe in some cases (Kahl et al., 2014; Weidner et al., 2015). Umbralisib/Ukoniq, an oral inhibitor of PI3Kδ and CK1-ε, was granted accelerated approval by the FDA for the treatment of MCL and FL in adults based on promising results from clinical trials but was later withdrawn from the market due to safety concerns (e.g., serious diarrhea or non-infectious colitis) (Dharmamoorthy et al., 2022). Copanlisib/BAY 80–6,946/Aliqopa, a highly selective intravenous pan-class I PI3K inhibitor with preferential inhibitory activity against the PI3Kα and PI3Kδ isoforms, was approved by the FDA for the treatment of relapsed FL and was further evaluated in clinical trials for several subtypes of non-Hodgkin’s lymphomas (Munoz et al., 2021). Diarrhea is also one of the most commonly reported treatment-emergent adverse events (TEAEs), occurring in 40.5% of patients with indolent and aggressive lymphoma in the phase II study (Dreyling et al., 2017). Duvelisib is the first FDA-approved oral dual inhibitor of PI3Kδ and PI3Kγ, with diarrhea reported as the most frequent any-grade adverse event (AE) (47%) and the most frequent grade ≥3 AE (12%) (Wang et al., 2023). The major mechanism of GI toxicity caused by PI3Kδ inhibitors is reported to be associated with the inhibition of B-cell differentiation through immune dysregulation of Tregs, leading to intestinal injury (Louie et al., 2015).

Overall, the clinical development of most PI3Kis has been discontinued from further development considering risk/benefit evaluations. Therefore, it is necessary to develop more specific inhibitors for individual isoforms of PI3K with improved toxic effect profiles, and comprehensively understanding the underlying mechanism of gastrointestinal toxicity is important for the clinical safety application of PI3Kis. Amdizalisib (HMPL-689) is a highly selective and potent PI3Kδ inhibitor with a different molecular structure discovered by HUTCHMED Limited. Preclinical data showed that amdizalisib was a potential best-in-class small-molecule inhibitor with high potency, favorable pharmacokinetics, and a drug safety profile. In this study, we conducted a battery of absorption, distribution, metabolism, and excretion (ADME) studies for amdizalisib, and finally, an allometric scaling (AS) approach was performed in order to estimate the total systemic clearance (CL) and volume of distribution (V_ss_) of amdizalisib in humans. We also analyzed the possible mechanism to lower the incidence of gastrointestinal toxicity for amdizalisib from a pharmacokinetic perspective.

## 2 Materials and methods

### 2.1 Chemicals and reagents

Amdizalisib (unlabeled) and an internal standard (HM5025815, a structure analog of amdizalisib) were provided by the Department of Medicinal Chemistry and the Department of Pharmaceutical Sciences at HUTCHMED Limited. Preclinical animals were purchased from standard vendors in China with certificates of laboratory animal production and usage. The human colon adenocarcinoma (Caco-2) cell line was purchased from the Cell Resource Center, Shanghai Institutes for Biological Sciences (Shanghai, China). Transwell^®^ 24-well plates and rapid equilibrium dialysis (RED) inserts with base plates were provided by Corning Costar (Corning, NY, United States) and Thermo Fisher Scientific (Waltham, MA, United States), respectively. HPLC-grade acetonitrile, isopropanol, ethyl acetate, ethanol, and methanol were purchased from Thermo Fisher Scientific (Waltham, MA, United States). Glucose-6-phosphate (G-6-P), glucose-6-phosphate dehydrogenase (G-6-PD), nicotinamide adenine dinucleotide phosphate (NADP), and HPLC-grade formic acid (FA) were obtained from Sigma-Aldrich (St. Louis, MO, United States). Dimethyl sulfoxide (DMSO), magnesium chloride (MgCl_2_), and ethylene diamine tetra-acetic acid (EDTA) were supplied by Sinopharm Chemical Reagent Co., Ltd (Shanghai, China). Substrates and inhibitors of CYP isoforms like acetaminophen, phenacetin, diclofenac, 4′-hydroxy-diclofenac, dextromethorphan, dextrorphan, 6β-hydroxy-testosterone, midazolam, 1′-OH midazolam, diazepam, 7-hydroxycoumarin, probenecid, erythromycin, verapamil, and nefazodone were supplied by Sigma-Aldrich (St. Louis, MO, United States). Testosterone was supplied by Acros Organics (Morris Plains, NJ, United States). Fetal bovine serum (FBS), 1X high-glucose Dulbecco’s Modified Eagle Medium (DMEM), 0.05% (1X) trypsin–EDTA solution, 1X Hank’s balanced salt solution (HBSS), 1 M (100X) N-2-hydroxyethylpiperazine-N′-2-ethanesulfonic acid (HEPES), 100X penicillin–streptomycin solution, 100 mM (100X) sodium pyruvate, 200 mM (100X) L-glutamine, and 100X minimum essential medium-non-essential amino acid (MEM-NEAA) solution were obtained from Gibco (Invitrogen, Carlsbad, CA, United States). Blank plasma from Institution of Cancer Research (ICR) mice and Sprague–Dawley (SD) rats was prepared in-house. Blank beagle dog plasma was obtained from the Suzhou Drug Safety Evaluation and Research Center (Suzhou, China). Blank cynomolgus monkey plasma was purchased from Labcorp Co. Ltd. (Shanghai, China). Blank human plasma was donated by staff at HUTCHMED Limited. Liver microsomes of humans, dogs, rats, mice, and male monkeys were purchased from Life Technologies (Durham, NC, USA), and those of female monkeys were supplied by the Research Institute for Liver Diseases Co., Ltd (Shanghai, China). Deionized water was prepared by the Millipore Gradient Water Purification System (Bedford, MA, United States) in-house. All other chemicals/reagents were of research grade and used without further purification.

### 2.2 *In vivo* ADME studies

#### 2.2.1 Dosing formulations

For oral administration in mice and monkeys, a homogenous suspension formulation was prepared using 0.5% CMC-Na (pH 2.1, adjusted by hydrochloric acid). For oral administration in rats and dogs, a homogenous suspension formulation was prepared using 0.5% CMC-Na. Amdizalisib was administered to mice and rats at 10 mL/kg body weight and dogs and monkeys at 2 and 5 mL/kg body weight, respectively. For intravenous administration in rodents, dogs, and monkeys, a solution formulation consisting of 10% Solutol HS 15, 10% ethanol, and 80% physiological saline was used. The volume of intravenous administration was 10, 4, 1, and 1 mL/kg for mice, rats, dogs, and monkeys, respectively. All intravenous and oral dose formulations were analyzed by liquid chromatography coupled with tandem mass spectrometry (LC-MS/MS) to confirm test article concentration and determine formulation stability.

#### 2.2.2 *In vivo* pharmacokinetic studies

The pharmacokinetic experiments in rodents were approved by the HUTCHMED Animal Care and Use Committee with approval numbers: HPD-113 (ICR mouse) and HPD-105 (SD rat). Animals were quarantined in a controlled environment within the Animal Resource Unit for a period of several days with 12:12 h light: dark cycles. Animals had free access to food, and water was allowed *ad libitum*. The animal experiments performed on dogs and monkeys were outsourced and conducted by Labcorp Co. Ltd (Shanghai, China). The blood samples were collected with sodium heparin as an anticoagulant. The plasma samples were obtained from blood samples by centrifugation and stored at −80°C until analysis.

The male ICR mice (26.8–31.2 g) were purchased from Shanghai SLAC Laboratory Animal Co., Ltd (Shanghai, China). A total of eighteen mice were divided into intravenous (IV) and oral (PO) administration groups, with nine animals in each group. The mice were fasted overnight with free access to water before dosing. Mice in the IV group were intravenously treated with 2.5 mg/kg amdizalisib, and mice in the PO group were orally treated with 10 mg/kg amdizalisib. In both groups, the blood samples were collected at pre-dose (0), 5, 15, 30 min, 1, 2, 4, 6, 8, 12, and 24 h after treatment. After the mice were anesthetized with isoflurane, approximately 100 μL of whole blood was collected through the retro-orbital sinus and transferred to a 1.5 mL tube containing sodium heparin. The gathered blood samples were kept on ice until centrifugation. Plasma samples were harvested by centrifugation at 1,500 *g* for 10 min at 4°C.

The SD rats (173–217 g, male: female = 1:1) were supplied by Shanghai SLAC Laboratory Animal Co., Ltd (Shanghai, China). For the single-dose PK study, twenty-four rats were divided into four groups, with six rats (male: female = 1:1) in each group. After fasting overnight, one group received intravenous administration of 5 mg/kg amdizalisib, and the other three groups received oral administration of 1, 5, and 25 mg/kg amdizalisib, respectively. Blood samples were collected at the following time points: pre-dose (0), 2, 5, 15, and 30 min and 1, 2, 4, 6, 8, 12, 24, and 48 h post IV dose; pre-dose (0), 15, and 30 min and 1, 2, 3, 4, 5, 6, 8, 10, 12, and 24 h for the 1 mg/kg post-PO dose; pre-dose (0), 15, and 30 min and 1, 2, 3, 4, 5, 6, 8, 10, 12, 24, and 48 h for the 5 mg/kg post-PO dose; and pre-dose (0), 15, and 30 min and 1, 2, 3, 4, 5, 6, 8, 10, 12, 24, 48, and 72 h for the 25 mg/kg post-PO dose, respectively. After rats were anesthetized with isoflurane, approximately 150 μL of whole blood was collected through the retro-orbital sinus and transferred to a 1.5 mL tube containing sodium heparin. The gathered blood samples were kept on ice until centrifugation. Plasma samples were harvested by centrifugation at 1,500 *g* for 10 min at 4°C.

For the tissue distribution study of amdizalisib, 33 SD rats (170–220 g, 15 males and 18 females) were purchased from Shanghai SLAC Laboratory Animal Co., Ltd (Shanghai, China). Rats were divided into seven groups according to the sampling time points, with six rats (male: female = 1: 1) in each group, except that Group 1 h used three male rats and Group 2 h and Group 72 h used three female rats, respectively. Rats were fasted overnight with free access to water before dosing. Each rat was orally administered 5 mg/kg amdizalisib with a dosing volume of 10 mL/kg. Plasma and tissue samples were collected at 0.5, 1, 6, 24, and 48 h after dosing for male rats and at 0.5, 2, 6, 24, 48, and 72 h after dosing for female rats. After rats were anesthetized with isoflurane, approximately 250 μL of whole blood was collected through the ophthalmic vein and centrifuged at 1,500 *g* under 4°C for 10 min to obtain plasma samples. After blood collection, the rats were sacrificed, and 18 tissues, namely, brain, breast, skin, fat, testis, ovary, bladder, pancreas, skeletal muscle, heart, lung, kidney, liver, stomach, small intestine, colon, spleen, and spinal cord, were removed from the rats at designated time points. These tissues were washed with saline and dried with filter paper. Aliquots of tissues were accurately weighed and then homogenized in 50% (v/v) methanol/water (10 mL/1 g tissue). For the excretion study of amdizalisib, [^14^C] amdizalisib was synthesized by Curachem, Inc. (Cheongju-si, Korea). Twelve rats (207–277 g) were divided into the bile-duct cannulation (BDC) group and the bile-duct intact (BDI) group, with six rats (male: female = 1:1) in each group. Both BDC rats and BDI rats orally received a single dose (5 mg/kg, 100 μCi/kg) of [^14^C] amdizalisib. Urine and feces were collected at pre-dose (0) and 24 h through 168 h post-dose (the first 24 h interval split into 0–8 and 8–24 h for urine) for BDI rats. For BDC rats, urine and feces were collected at pre-dose (0) and 72 h post-dose (the first 24 h interval split into 0–8 and 8–24 h for urine), and bile was collected at pre-dose (0), 0–4, 4–8, 8–24, 24–48, and 48–72 h post-dose. Cage-washing fluids were collected at 24 h intervals until the last sampling time point for both groups. Another BDI rat was orally administered a blank vehicle (0.5% CMC-Na, 0.5 g/100 mL), and urine and feces were collected 24 h post-dose. The weight of the collected samples was recorded. Samples were stored in a −20°C freezer until analysis.

Twelve beagle dogs (6–10 kg, male: female = 1:1) purchased from Beijing Marshall Biotechnology Co. Ltd. (Beijing, China) were enrolled in a three-period self-control cross-over study with a washout time of 1 week. The effect of food intake on the pharmacokinetics of amdizalisib was also evaluated in this study. In Period 1 and Period 2, dogs were fasted overnight until dosing. In Period 1, dogs in group 1 received an IV dose of 0.5 mg/kg amdizalisib, and dogs in group 2 were orally given 0.5 mg/kg amdizalisib. Blood samples were collected at pre-dose (0), 2, 5, 15, and 30 min and 1, 2, 3, 4, 6, 8, 10, 12, 24, and 32 h post-dose for IV dosing and at pre-dose (0), 15, and 30 min and 1, 1.5, 2, 3, 4, 5, 6, 8, 10, 12, 24, and 32 h post-dose for PO dosing. In Period 2, dogs in groups 1 and 2 were orally given 3.5 and 25 mg/kg amdizalisib, respectively. Blood samples were collected at pre-dose (0), 15, and 30 min and 1, 1.5, 2, 3, 4, 5, 6, 8, 10, 12, 24, 32, and 48 h post-dose for group 1 and at pre-dose (0), 15, and 30 min and 1, 1.5, 2, 3, 4, 5, 6, 8, 10, 12, 24, 32, 48, and 72 h post-dose for group 2. In Period 3, dogs in group 2 were fasted overnight but fed for 30 min just before orally dosing 25 mg/kg of amdizalisib, and the blood sampling time points were the same as those at the same dose level in Period 2. Approximately 1.0 mL of blood was collected per time point from the jugular vein using a 2 mL injector and transferred to a 3 mL tube that contained sodium heparin. The collected blood samples were centrifuged at 3,500 g for 5 min at 4°C to collect plasma within 30 min after sample collection. Cynomolgus monkeys (3.3–4.6 kg, male: female = 1:1) purchased from Huazhen Laboratory Animal Breeding Center (Guangzhou, China) were enrolled in a two-period self-control cross-over study with a washout time of 1 week. Animals were fasted overnight and for approximately 4 h post-dose. In Period 1, six monkeys were intravenously administered 1 mg/kg amdizalisib, and in Period 2, the same six monkeys were orally administered 5 mg/kg amdizalisib. Blood samples were collected at pre-dose (0), 2, 5, 15, and 30 min and 1, 2, 4, 6, 8, 12, 24, and 48 h post IV dose and at pre-dose (0), 5, 15, and 30 min and 1, 2, 3, 4, 6, 8, 12, 24, and 48 h post PO dose. At each time point, approximately 1.0 mL of blood was collected from the femoral vein and transferred into an individual tube that contained sodium heparin. The blood samples were centrifuged at 3,500 *g* for 5 min at 4°C to harvest plasma.

### 2.3 *In vitro* ADME studies

#### 2.3.1 Caco-2 transport

The bidirectional permeability and absorption mechanism of amdizalisib were evaluated across Caco-2 cell monolayers. The passage number of cells used in this study was 39. Non-specific binding to the device surfaces and the aqueous solubility of test compounds were tested before the transport experiment. Caco-2 cells were cultured in a 24-well Transwell^®^ Insert at 37°C in an atmosphere of 5% CO_2_ and 90% relative moisture for 21 days to form monolayers. The integrity of the monolayer was estimated by measuring transepithelial electrical resistance (TEER). Only the monolayers with TEER over 150 Ω were used for the studies. The culture medium was 1× high-glucose Dulbecco’s Modified Eagle Medium (DMEM) containing 10% FBS (v/v), 1% MEM-NEAA (v/v), 2 mM L-glutamine, 1 mM sodium pyruvate, 10 mM HEPES, 100 units/mL penicillin, and 100 μg/mL streptomycin. The transport medium buffer was 1× HBSS solution containing 10 mM HEPES and 1.2% (v/v) DMSO. The bidirectional transport studies were initiated by adding amdizalisib to either the apical (A) or basolateral (B) side of the Transwell^®^ Insert. After 30 min of pre-incubation with blank transport medium buffer, concentration-dependent transport of amdizalisib was evaluated at 5–80 μM, and time-dependent transport of amdizalisib was assessed at 10 µM after incubation for 30–150 min. Atenolol (10 µM) and metoprolol (10 µM) were used as a low-permeable control and a high-permeable control in this study, respectively. After incubation, samples from both the donor and receiver sides were collected, and the concentrations of amdizalisib and control compounds were determined by LC-MS/MS. The apparent permeability coefficient (P_app_) and the efflux ratio (ER) of P_app_ were calculated according to the following equations:

$$P_{\mathrm{app}\;}{\left(10\right.}^{‐6\;}\mathrm{cm}/\left.\sec\right)=\frac{V_{r}\times C_{r,t}}{S\times t\times C_{d,0}},$$

$${\text{Efflux}\;r\text{atio}\;\left(\text{ER}\right)=P}_{\text{app},B‐A\;}{/\;P}_{\text{app},A‐B},$$

where V_r_ is the donor volume (0.3 mL for A-B; 1 mL for B-A); C_d, 0_ is the measured concentration of amdizalisib or controls on the donor side at time zero; C_r, t_ is the measured concentration of amdizalisib or controls on the receiver side at time t; S is the surface area of the cell monolayer (0.33 cm^2^); and t is the incubation time (sec).

The inhibitory effects of amdizalisib on P-gp and BCRP activities were also investigated in Caco-2 cell monolayers. The experimental conditions were similar to those of the permeability study. Digoxin (5 µM), a substrate of P-gp, was incubated in the absence or presence of amdizalisib (0.1–80 µM) or the absence or presence of LY335979 (1 μM, a specific and strong P-gp inhibitor) and Ko143 (10 μM, a negative or weak P-gp inhibitor). Estrone-3-sulfate (E3S, 2.5 µM), a substrate of BCRP, was incubated in the absence or presence of amdizalisib (0.1–80 µM) or the absence or presence of Ko143 (1 μM, a specific and strong BCRP inhibitor) and LY335979 (1 μM, a negative or weak BCRP inhibitor). After 60 min of incubation, samples on both sides were collected, and concentrations of digoxin and E3S were determined by LC-MS/MS. P_app_ and ER were calculated, and the equation for calculating the percentage of substrate transport inhibition was shown as follows:

$$\%\text{ transport inhibition}=\left(1-\frac{P_{\text{app},B-A,C+I}-P_{\text{app},A-B,C+I}}{P_{\text{app},B-A,C}-P_{\text{app},A-B,C}}\right)\times 100\%,$$

where P_app, C+I_ is the P_app_ value of the substrate in the presence of amdizalisib or transporter inhibitors and P_app, C_ is the P_app_ value of the substrate in the absence of amdizalisib or transporter inhibitors.

#### 2.3.2 Plasma protein binding and whole blood-to-plasma ratio

Rapid equilibrium dialysis (RED), which is the most common approach for the evaluation of plasma protein binding, was used to evaluate the ability of amdizalisib to bind the plasma proteins. Stock solutions of amdizalisib were spiked into five different species (mice, rats, dogs, monkeys, and humans) plasma to achieve the designated concentrations (0.1, 1, and 20 μM). In addition, 300 μL of plasma samples containing amdizalisib (0.1, 1, or 20 μM) and 500 µL of dialysis buffer (containing 100 mM sodium phosphate buffer and 150 mM NaCl, pH = 7.4) were spiked into the donor and receiver cell chambers of the equilibrium dialysis device, respectively. The plate containing plasma and buffer was equilibrated for 6 h in a 37°C water shaking bath. After incubation, aliquots of buffer and plasma (20 µL) on both sides were collected and precipitated using organic solvents. The concentrations of amdizalisib were determined by LC-MS/MS. Plasma samples containing amdizalisib at each concentration level were prepared and incubated in a water bath at 37°C for 6 h to evaluate the plasma stability of the test compound. The post-dialysis recovery of amdizalisib was also measured. Plasma protein binding fraction (PPB%), post-dialysis recovery (Recovery%), and post-dialysis stability (Stability%) were calculated using the following equations:

$${\text{PPB}\%=(C}_{d\;}{‐C}_{r})\;{/\;C}_{d}\times 100,$$

$$\text{Recovery}\%=\left(C_{r}\times 500+C_{d}\times 300\right)\;/\;\left(\;C_{6}\times 300\right)\times 100,$$

$$\text{Stability}\%=C_{6}\;{/\;C}_{0}\times 100,$$

where C_d_ or C_r_ is the determined concentration of amdizalisib in the donor or receiver cell chamber, respectively. C_0_ or C_6_ is the determined concentration of amdizalisib in plasma samples without incubation or with incubation for 6 h.

*In vitro* blood distribution of amdizalisib was investigated in the whole blood of rats, dogs, and humans according to the published method with slight modifications (Yu et al., 2005). Amdizalisib was spiked into the fresh whole blood from different species and the corresponding fresh plasma (reference control plasma) to achieve a final concentration of 1 μM. The whole blood and plasma samples were incubated at 37°C for up to 1 h. Serial samples (150 µL) were collected at different time points (0, 10, and 60 min) and transferred into new tubes. The whole blood samples were then centrifuged at 1700 *g* for 15 min to harvest plasma. After protein precipitation, the concentration of amdizalisib in two types of plasma samples was determined by LC-MS/MS. The whole blood-to-plasma partition coefficient (R_B_) and the unbound fraction of amdizalisib in blood (f_uB_) were calculated as follows:

$$R_{B}=C_{\text{PL}\;}^{\text{REF}}{/\;C}_{\text{PL}},$$

$$f_{\text{uB}\;}{=f}_{\text{up}\;}{/\;R}_{B\;},$$

where C^REF^_PL_ is the concentration of amdizalisib in the reference control plasma; C_PL_ is the concentration of amdizalisib from the plasma equilibrating with RBC (red blood cells); and f_up_ is the unbound fraction of amdizalisib in plasma, which is obtained from the plasma protein binding study.

#### 2.3.3 Metabolic stability

*In vitro* metabolic stability of amdizalisib in liver microsomes from different species (mice, rats, dogs, monkeys, and humans) was conducted in triplicate at 1 µM. The metabolic reaction mixture consisted of liver microsomes (0.5 mg/mL) or flavin-containing monooxygenase (FMO)-deactivated liver microsomes (0.5 mg/mL), 1 μM amdizalisib, 3 mM MgCl_2_, and 1 mM EDTA in 50 mM potassium phosphate buffer at pH 7.4. FMO was deactivated by heating liver microsomes at 45°C for 5 min. Following pre-incubation at 37°C for 10 min, the NADPH-regenerating system (1 mM NADP, 5 mM G-6-P and 1 Unit/mL G-6-PD) was spiked into the metabolic reaction mixture to start the reaction and terminated at 0, 5, 15, 30, 60, and 120 min by the addition of 125 μL cold acetonitrile containing internal standard (IS). The terminated incubation mixtures were centrifuged at 1,500 *g* for 10 min, and then the supernatant (70 µL) was diluted with 70 µL deionized water before injection into the LC-MS/MS system for analysis.

The peak area ratios of the analyte/IS were converted to a percentage of the drug remaining using the peak area ratio at time zero as 100%. The natural logarithm of the percentage of the drug remaining was plotted against the incubation time, and the slope of the linear regression (-k) was converted to *in vitro* half-lives (t_1/2_) using the following equation:

$$In\;vitro{\;t}_{1/2}=0.693\;/\;k,$$

*In vitro* intrinsic clearance (CL_int,_ _*in vitro*_) and scaled *in vivo* intrinsic clearance (CL_int,_ _*in vivo*_) values (in units of mL/min/kg) were further estimated according to literature methods (Kuhnz and Gieschen, 1998; Obach, 1999; Zientek et al., 2010) without correction for the unbound fraction of amdizalisib in blood and based on the following assumptions: (1) metabolic clearance is the major mechanism of clearance; (2) liver is the major organ of clearance; and (3) oxidation is the major metabolic pathway.

$${\text{CL}}_{\mathrm{int},in\;vitro}=k\;/\;C_{\text{microsomes}\;},$$

$${\text{Scaled}\;\text{CL}}_{\mathrm{int},in\;vivo}={\text{CL}}_{\mathrm{int},in\;vitro}\times \text{microsomes yield}\times \text{liver weight},$$

where C_microsomes_ means the experimental protein concentration (0.5 mg/mL) in the incubation system. A microsome yield (45 mg protein/g liver) of all species was used, with the liver weight values for mice, rats, dogs, monkeys, and humans approximately being 87.5, 40, 32, 30, and 25.7 g/kg body weight, respectively (Davies and Morris, 1993).

Then, the scaled *in vivo* intrinsic clearance was used to predict the systemic clearance (equal to the hepatic clearance) using the well-stirred venous equilibration model (Houston, 1994).

$${\mathrm{CL}}_{\mathrm{sys}}{=\mathrm{CL}}_{\mathrm{int}\;\mathrm{in}\;\mathrm{vivo}\;}\times {\;Q}_{H}{\;/\;(Q}_{H}{+\mathrm{CL}}_{\mathrm{int}\;\mathrm{in}\;\mathrm{vivo}}),$$

where Q_H_ is the hepatic blood flow: 90 mL/min/kg for mice, 55.2 mL/min/kg for rats, 30.9 mL/min/kg for dogs, 43.6 mL/min/kg for monkeys, and 20.7 mL/min/kg for humans (Davies and Morris, 1993).

#### 2.3.4 CYP inhibition

Reversible inhibition potentials of amdizalisib against eight major CYP isoforms (CYP1A2, 2B6, 2C8, 2C9, 2C19, 2D6, 2E1, and 3A4/5) were assessed in human liver microsomes (HLMs). Inhibitory activity was evaluated by incubating different concentration levels of amdizalisib (0.4–50 µM for CYP2B6 and 0.08–50 µM for the rest) and/or reference inhibitors with HLMs. The markers used to determine the activities of CYP isoforms were phenacetin O-deethylation for CYP1A2 (probe substrate: 50 µM phenacetin), bupropion hydroxylation for CYP2B6 (probe substrate: 70 µM bupropion), paclitaxel 6α-hydroxylation for CYP2C8 (probe substrate: 10 µM paclitaxel), diclofenac 4′-hydroxylation for CYP2C9 (probe substrate: 5 µM diclofenac), S-(+)-mephenytoin 4′-hydroxylation for CYP2C19 (probe substrate: 20 µM S-(+)-mephenytoin), dextromethorphan O-demethylation for CYP2D6 (probe substrate: 6 µM dextromethorphan), chlorzoxazone 6′-hydroxylation for CYP2E1 (probe substrate: 70 µM chlorzoxazone), and testosterone 6β-hydroxylation and midazolam 1′-hydroxylation for CYP3A4/5 (probe substrate: 45 µM testosterone and 5 µM midazolam). The CYP inhibition reaction mixtures contained HLMs (0.5 mg/mL for CYP2C19 and 0.2 mg/mL for the rest of the CYP isoforms), probe substrates, amdizalisib or positive inhibitors, the NADPH-regenerating system (the same as above), MgCl_2_ (3 mM), EDTA (1 mM), pure water, and 50 mM potassium phosphate buffer in a total volume of 125 μL. Alpha-naphthoflavone (0.04 µM), 2-phenyl-2-(1-piperidinyl)propane (5 µM), quercetin (1 µM), sulfaphenazole (0.5 µM), ticlopidine (4 µM), quinidine (0.2 µM), diethyldithiocarbamate (20 µM), and ketoconazole (0.05 µM) were used as positive inhibitors of CYP1A2, 2B6, 2C8, 2C9, 2C19, 2D6, 2E1, and 3A4, respectively. The vehicle control was prepared in the same composition as inhibitor solutions but free of inhibitors. The concentrations of probe substrates were close to K_m_ values that were determined in-house and within the range recommended by the FDA (Huang et al., 2007). Reaction mixtures were incubated at 37°C for 5, 20, or 30 min, depending on the probe substrates of CYP isoforms. The reactions were terminated by adding cold acetonitrile containing IS, and the terminated incubation mixtures were centrifuged at 3,000 *g* for 10 min before LC-MS/MS analysis. The remaining activity of each CYP isoform was calculated by dividing the enzyme activity of amdizalisib or the positive inhibitor group by the enzyme activity of the vehicle control group.

The inhibitor concentrations along with the corresponding remaining enzyme activities were analyzed by the sigmoid and inhibitory effect E_max_ model using Phoenix WinNonlin (Certara USA Inc., Princeton, United States) to calculate the concentration required to achieve 50% inhibition (IC_50_). If the remaining CYP activity in the presence of the highest tested concentration of amdizalisib was greater than 50.0%, IC_50_ was expressed as greater than the highest tested concentration.

The procedure of time-dependent CYP inhibition was similar to that used for CYP reversible inhibition, as mentioned above, except that a pre-incubation step was added. The mixtures containing amdizalisib (10 μM) or positive inhibitors in the absence and presence of the NADPH-regenerating system were pre-incubated at 37°C for 30 min before moving on to the next step. Erythromycin, verapamil, and nefazodone at 10 μM were selected as weak, moderate, and strong inhibitors on CYP3A4/5, ticlopidine at concentrations of 0.4 μM and 4 μM was selected as a positive inhibitor on CYP2B6 and CYP2C19, respectively, and phenelzine sulfate at 200 μM was used as a positive inhibitor on CYP2C8. The probe substrates for CYP1A2, 2B6, 2C8, 2C9, 2C19, 2D6, and 3A4/5 were phenacetin (250 µM), bupropion (450 µM), amodiaquine hydrochloride (15 µM), diclofenac (25 µM), S-(+)-mephenytoin (200 µM), dextromethorphan (30 µM), testosterone (225 µM), and midazolam (30 µM), respectively. Another appropriate volume of the NADPH-regenerating system and probe substrate were added to initiate the CYP isoform enzyme activity test step. For this step, the incubation period was 10 min, except for CYP2B6, CYP2C8, and CYP3A4/5 (probe substrate: midazolam), which was 3 min to make sure the reaction was under initial linear rates. After incubation for the designated time periods at 37°C, the ice-cold acetonitrile containing IS was added to quench the reaction. The samples were centrifuged at 1,500 *g* at 4°C for 10 min, and the supernatant fractions were analyzed by LC-MS/MS. The activity loss of each CYP isoform was calculated as follows:

$$\text{The activity loss}\left(\%\right)=100\times [{\left(A_{\text{amdizalisib}\;\text{or}\;\text{positive}\;\text{inhibitors}\;}/A_{\text{vehicle}}\right)}_{‐\text{NADPH}}-{\left(A_{\text{amdizalisib}\;\text{or}\;\text{positive}\;\text{inhibitors}\;}/\;A_{\text{vehicle}}\right)}_{+\text{NADPH}},$$

where A represents measured enzyme activities in the presence and absence of amdizalisib or positive inhibitors and NAPDH. (A_inactivator_/A_vehicle_)_-NADPH_ represents the enzyme activity remaining due to the inhibition by the diluted inactivator in the enzyme activity test step, and (A_inactivator_/A_vehicle_) _+NADPH_ represents the remaining enzyme activity due to pre-incubation loss plus inhibition by the diluted inactivator in the enzyme activity test step.

#### 2.3.5 CYP induction

Induction potential on CYP1A2, 2B6, 3A4, 2C8, 2C9, and 2C19 activities by amdizalisib was examined in cryopreserved human hepatocytes (BioIVT, Baltimore, MD, USA) from three donors. Donor BXW (female, Caucasian, 73 years old), XSM (female, Spanish, 59 years old), and NFX (male, Caucasian, 36 years old) were used in the CYP1A2, 2B6, and 3A4 induction assays. Donors GKJ (male, Caucasian, 52 years old), ZEY (male, Caucasian, 62 years old), and WKF (female, Caucasian, 59 years old) were used in the CYP2C induction assay. In brief, three separate lots of cryopreserved human hepatocytes were treated once daily with vehicle (0.1% DMSO), amdizalisib (1.00, 3.00, 10.0, 20.0, and 30.0 µM for CYP1A2, 2B6, and 3A4/5, 0.3, 3, and 20 µM for CYP2C8, 2C9, and 2C19) or one of the following positive inducers: 50 μM omeprazole (CYP1A2), 1,000 μM phenobarbital (CYP2B6), 10 μM rifampin (CYP3A4/5), and 25 µM rifampin (CYP2C8, 2C9, and 2C19). Flumazenil (25 µM) was used as the negative control treatment for CYP2C8, 2C9, and 2C19. The probe substrates for CYP1A2, 2B6, 3A4, 2C8, 2C9, and 2C19 were phenacetin (250 µM), bupropion (500 µM), midazolam (25 µM), amodiaquine (15 µM), diclofenac (40 µM), and S-(+)-mephenytoin (80 µM), respectively. Following 48 h of incubation, an appropriate volume of incubation medium was collected to determine the metabolites of each substrate, including acetaminophen, hydroxyl bupropion, 1′-hydroxy midazolam, N-desethyl amodiaquine, 4′-hydroxy diclofenac, and 4′-hydroxy mephenytoin. Enzyme activity for each CYP isoform was reported as a fold change over vehicle control and the percent of positive control (detailed calculation in Supplementary 1.1). Hepatocytes were harvested with a lysis buffer from RNeasy 96 Kit (Qiagen Inc., Germantown, MD, USA) to isolate RNA, which was analyzed by real-time quantitative polymerase chain reaction (RT-PCR). The effect of amdizalisib on CYP1A2, 2B6, 3A4/5, 2C8, and 2C9 mRNA levels was reported as the percent of positive control (detailed calculation in Supplementary 1.1). E_max_ (the maximal observed induction) and EC_50_ (the concentration that supports half-maximal induction) were estimated by non-linear regression of fold increase in CYP mRNA expression *versus* concentration plots. The amdizalisib concentrations at different time points after the last incubation (CYP2C induction assay) were measured by LC-MS/MS, and the percentage of amdizalisib remaining was determined on the last day of treatment (CYP1A2, 2B6, and 3A4 induction assay).

#### 2.3.6 Transporter inhibition

The inhibition of amdizalisib on substrate uptake mediated by OATP1B1, OATP1B3, OAT1, OAT3, OCT2, MATE1, and MATE2-K was explored in stable transfected HEK293 cell lines expressing these human drug transporters. Pre-incubation solutions containing amdizalisib (0.3, 1, 3, 10, 30, and 100 μM) or the positive control inhibitors (or transport buffer containing an equal volume of vehicle control) were added to the cell plate and incubated at 37°C for 30 min. The positive control inhibitor for OAT1 and OAT3 was probenecid (0.1, 0.3, 1, 3, 10, 30, and 100 μM); for OCT2, it was verapamil (0.3, 1, 3, 10, 30, 100, and 300 μM); for OATP1B1 and OATP1B3, it was rifampicin (0.01, 0.03, 0.1, 0.3, 1, 3, and 10 μM); and for MATE1 and MATE2-K, it was pyrimethamine (0.001, 0.003, 0.01, 0.03, 0.1, 0.3, and 1 μM). The pre-incubation solutions were removed, and incubation solutions containing the inhibitors and probe substrates (or transport buffer containing an equal volume of vehicle control and probe substrates) were added and incubated at 37°C for 2 min (5 min for OATP1B3). The probe substrate for OAT1 was 4-aminohippuric acid (10 μM), for OAT3 was estrone 3-sulfate (10 μM), for OCT2 was metformin (100 μM), for OATP1B1 was β-estradiol 17-(β-D-glucuronide) (5 μM), for OATP1B3 was β-estradiol 17-(β-D-glucuronide) (10 μM), and for MATE1 and MATE2-K was tetraethylammonium (10 μM). After incubation, cells were washed three times with ice-cold transport buffer (25 mM HEPES, pH 7.4, pH 8.0 for overexpressing MATE1 and MATE2-K HEK293 cells) and lysed in distilled water. The concentrations of probe substrates were determined by LC-MS/MS, and a BCA Kit (Solarbio ^®^Life Sciences, Beijing, China) was used to determine the protein content in the cell lysate. The uptake rate (pmol/mg protein/min), the percentage transported (%) for substrate, and the percentage inhibition (%) were calculated according to the following equations:

$$\mathrm{Uptake}\;\mathrm{rate}\;\left(\mathrm{pmol}/\mathrm{mg}\;\mathrm{protein}/\min\right)=C\;/\;\left(P\times T\right),$$

$$\text{Percentage transported }\left(\%\right)=\frac{{\text{Uptake}\;\text{rate}}_{\text{test}\;\text{compound}}\;‐\;{\text{Uptake}\;\text{rate}}_{\text{passive}}}{{\text{Uptake}\;\text{rate}}_{\text{vehicle}\;\text{control}}\;‐\;{\text{Uptake}\;\text{rate}}_{\text{passive}}}\times 100,$$

$$\text{Percentage inhibition }\left(\%\right)=100\;‐\text{ Percentage transported }\left(\%\right),$$

where C is the concentration of the drug in the cell lysate (nM), P is the protein concentration of the cell lysate (mg/mL), and T is the incubation time (minute). Uptake rate_test compound_ is the uptake rate of the substrate in the presence of the test compound. Uptake rate_vehicle control_ is the uptake rate of the substrate in the presence of the vehicle control. Uptake rate_passive_ is the uptake rate of the substrate in the presence of the positive inhibitor at the top test concentration.

The inhibitory potency of amdizalisib/positive inhibitors was further evaluated with their IC_50_ values (the concentration to exert 50% of the maximal inhibitory effect on transporter activity). Where appropriate, the calculated percentage transported (%) was plotted against nominal inhibitor concentrations and fitted using XLfit (4-parameter logistic model, equation 201: parameter C equivalent to IC_50_) to calculate IC_50_. When percentage inhibition (%) at the maximum concentration tested was lower than 50.0%, IC_50_ was indicated as > the maximum concentration.

### 2.4 Prediction of human pharmacokinetic parameters using allometric scaling

Translational prediction of human plasma total clearance (CL) and volume of distribution at steady state (V_ss_) was projected using the AS approach for different preclinical species (mice, rats, dogs, and monkeys). The mean value of unbound plasma fraction (f_u_) in animals was applied to transform pharmacokinetic parameters (V_ss_ or CL) to the corresponding free drug clearance (CL_u_) and free drug volume of distribution (V_ss,u_). The CL_u_ in preclinical species was scaled to CL_u_ in humans according to the following equations with consideration of the maximum lifespan potential (MLP) as a correction factor according to the rule of exponents (RoEs) (Mahmood and Balian, 1996), and V_ss,u_ in preclinical species was scaled to V_ss,u_ in humans simply based on BW according to the following equations. Both CL_u_ and V_ss,u_ in humans were converted to CL and V_ss_ using the mean value of f_u_ in humans.

$${\text{CL}}_{u}{\times \text{MLP}=a\times \text{BW}}^{b},$$

$${\log\;(\text{CL}}_{u}\times \text{MLP})=\log\;a+b\;\log\text{ BW},$$

$$V_{\text{ss},u}{=a\times \text{BW}}^{b},$$

$${\log\;(V}_{\text{ss},u})=\log\;a+b\;\log\text{ BW},$$

where BW represents the body weight of the standard species (0.02, 0.2, 10, 4, and 70 kg for mice, rats, dogs, cynomolgus monkeys, and humans, respectively), a represents allometric coefficients, and b is allometric exponents.

### 2.5 Quantitative sample analysis

A universal liquid chromatography-tandem mass spectrometry (LC-MS/MS, AB SCIEX API-4500, Foster City, CA) method was applied for exploratory purposes in mouse and monkey PK studies, and a validated bioanalytical method was used in the determination of amdizalisib concentrations in rat and dog PK studies. An aliquot of 20 µL of plasma or tissue homogenate samples was precipitated with 80 µL of acetonitrile containing 200 ng/mL IS (a structural analog of amdizalisib) and centrifuged at 20,800 g at 4°C for 10 min (Eppendorf 5810R, Hamburg, Germany) after vortexing for 2 min. The clear supernatant (70 µL) was mixed with 70 µL of deionized water before injection into the LC-MS/MS system for analysis. The lower limit of quantification (LLOQ) of amdizalisib was 1 ng/mL in rat and dog studies and 2.44 ng/mL in mouse and monkey studies. The quantification range of amdizalisib was 2.44–10,000 ng/mL in mouse and monkey studies and 1–1,000 ng/mL in rat and dog studies. The detailed, universal, and validated analytical methods are provided in Supplementary 1.2.

For the determination of drug-derived radioactivity in the excretion study in SD rats, urine (0.1 g), bile (0.025 g), and cage wash samples (1 g) were mixed well with scintillation solution (5 mL for urine and cage wash samples; 10 mL for bile samples) and directly analyzed by a liquid scintillation counter (LSC, Perkin Elmer, Waltham, MA, United States). An appropriate amount of isopropanol: water (1:1, v/v) was added to the feces samples and thoroughly homogenized. Pooled fecal homogenate (0.3 g) was combusted in a Sample Oxidizer 501 (RJ Harvey Instrument, Tappan, NY, USA), and the generated ^14^CO_2_ was trapped in a 15 mL scintillation solution and further analyzed for radioactivity by LSC using a Tri-Carb 3110 TR counter (Perkin Elmer, Waltham, MA, United States). The radioactive concentrations of urine, bile, feces, and cage washings were calculated with the counted disintegration per minute (DPM). The percentage of administered dose recovered (% dose recovered) was calculated using the following equation:

$$\%\text{ dose recovered}=\text{Total radioactivity }\left(\text{DPM}\right)\text{ in sample}/\text{ radioactivity }\left(\text{DPM}\right)\text{ in dosing formulation}\times 100.$$

An appropriate volume of samples from *in vitro* PK studies was diluted with appropriate matrices, followed by direct LC-MS/MS injection or protein-precipitated by acetonitrile before injection. The LC-MS/MS conditions were the same as the universal method or the validated method mentioned above.

### 2.6 Pharmacokinetic analysis

Pharmacokinetic parameters were calculated by a non-compartmental method using Thermo Kinetica^®^ (Version 5.1 SP1, Thermo Electron Corporation, Philadelphia, Pennsylvania, United States). The area under the plasma concentration–time curve from time zero to the time of the last measurable sample (AUC_0-t_) or the plasma concentration–time curve from time zero to infinity (AUC_0-inf_) was calculated using the linear trapezoidal method. Dose-normalized AUC and C_max_ (AUC/Dose and C_max_/Dose) were used to evaluate whether or not drug exposure increased dose proportionally. If the ratio of dose-normalized exposures (AUC/Dose and C_max_/Dose) between two dose levels was less than 2, the change in drug exposure was deemed dose-proportional. Absolute oral bioavailability (F_PO_%) was calculated using the following equation:

$$F_{\text{PO}\;}\left(\%\right){=[\text{Dose}\;\left(\text{IV}\right)\times \text{AUC}}_{0‐t\;\text{or}\;0‐\inf,\text{PO}}{\;/\;\text{Dose}\;\left(\text{PO}\right)\times \text{AUC}}_{0‐t\;\text{or}\;0‐\inf,\text{IV}}]\times 100.$$

## 3 Results

### 3.1 PK in mice, rats, dogs, and cynomolgus monkeys

**FIGURE 1:**
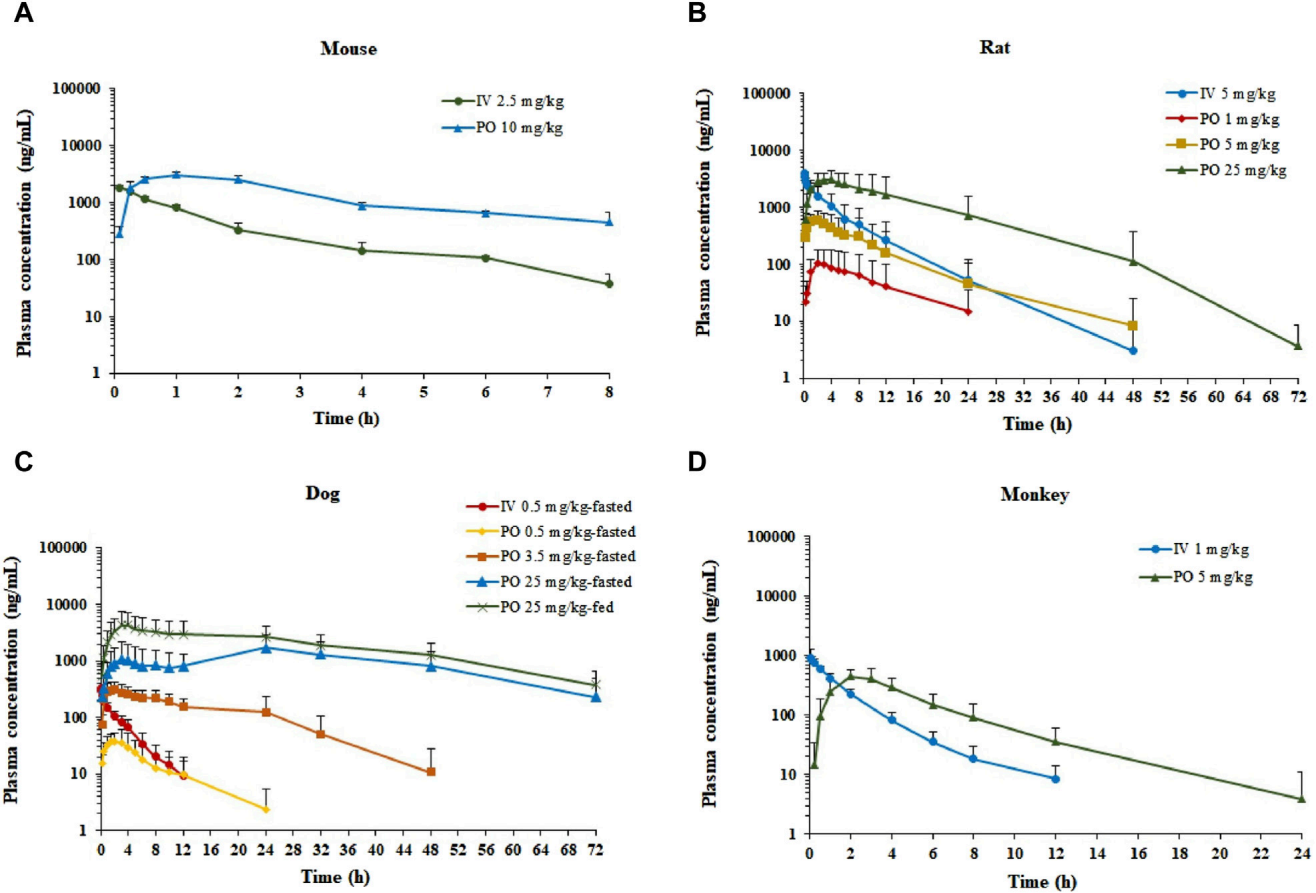
Plasma concentration–time profiles of amdizalisib after a single oral or intravenous dosing in mice, rats, dogs, and monkeys. **(A)** ICR mice (n = 3), **(B)** SD rats (n = 6), **(C)** beagle dogs (n = 6), and **(D)** cynomolgus monkeys (n = 6); all data are expressed as the mean ± standard deviation; IV, intravenous administration; PO, oral administration.

The semi-log plasma concentrations of amdizalisib *versus* time profiles are shown in Figure 1, and the derived PK parameters in ICR mouse, SD rat, beagle dog, and cynomolgus monkey plasma derived from non-compartmental analysis are presented in Table 1. All intravenous and oral dose formulations were determined to have 100% ± 10% recovery for solutions and 100% ± 15% recovery for suspensions.

**TABLE 1:** Pharmacokinetic parameters of amdizalisib after oral or intravenous administration to mice, rats, dogs, and monkeys (expressed as the mean ± standard deviation where applicable).

Animal species	Feeding condition	Route	Dose (mg/kg)	T_max_ (h)	C_max_ (ng/mL)	AUC_0-t_ (h·ng/mL)	AUC_0-inf_ (h·ng/mL)	V_ss_ (L/kg)	t_1/2_ (h)	MRT (h)	CL (mL/min/kg)	F_PO_ (%)
ICR mouse	Fasted	IV	2.5	-	-	2,705	2,831	1.87	2.06	2.12	14.7	-
		PO	10	1.00	3,080	10,945	13,684	-	4.16	4.91	-	101
SD rat	Fasted	IV	5	-	-	13,853 ± 8,804	13,880 ± 8,832	1.68 ± 0.153	3.61 ± 2.01	4.60 ± 2.85	9.12 ± 6.31	-
		PO	1	2.67 ± 1.75	115 ± 79.1	1,141 ± 1,310	1,320 ± 1,538	-	4.94 ± 3.35	7.90 ± 5.28	-	-
			5	1.50 ± 0.548	622 ± 257	6,038 ± 5,516	6,130 ± 5,520	-	5.11 ± 4.00	7.84 ± 5.51	-	39.8 ± 18.0
			25	4.17 ± 2.23	3,348 ± 1,117	52,403 ± 40,848	52,608 ± 40,748	-	5.62 ± 2.86	11.0 ± 6.61	-	-
Beagle dog	Fasted	IV	0.5	-	-	750 ± 272	772 ± 272	2.40 ± 0.354	3.02 ± 1.27	3.65 ± 1.29	11.8 ± 3.61	-
		PO	0.5	1.92 ± 0.665	42.4 ± 21.8	323 ± 232	359 ± 259	-	7.22 ± 6.55	9.66 ± 6.73	-	43.1 ± 31.0
			3.5	6.25 ± 9.11	347 ± 105	5,538 ± 2,416	5,658 ± 2,583	-	6.08 ± 1.48	13.8 ± 4.85	-	-
			25	15.0 ± 10.4	2,159 ± 1,070	66,480 ± 35,583	72,773 ± 42,635	-	13.9 ± 5.07	31.8 ± 10.2	-	-
	Fed	PO	25	7.00 ± 8.34	4,440 ± 3,071	1,34,308 ± 81295	145,323 ± 8,885	-	17.4 ± 2.49	30.2 ± 4.23	-	-
Cynomolgus monkey	Fasted	IV	1	-	-	1,486 ± 272	1,522 ± 297	1.45 ± 0.207	2.82 ± 0.469	2.20 ± 0.551	11.3 ± 2.36	-
		PO	5	2.00 ± 0.632	480 ± 147	2,331 ± 1,028	2,389 ± 1,050	-	3.10 ± 0.890	4.90 ± 1.00	-	30.8 ± 8.48

PK parameters were calculated based on the mean plasma concentrations for mice and individual plasma concentrations for rats, dogs, and monkeys. Dash (−) indicates not determined. F_PO_% refers to absolute oral bioavailability, which was calculated based on IV and PO exposures. The %AUC_extra_ was not above 20% in all preclinical species.

After a single IV dose, the clearance (CL) of amdizalisib in mice, rats, dogs, and cynomolgus monkeys was 14.7, 9.12, 11.8, and 11.3 mL/min/kg, accounting for 16.3, 16.6, 38.1, and 25.7% of the hepatic blood flow of the corresponding species (using species-specific hepatic blood flow of 90, 55, 31, and 44 mL/min/kg, respectively (Davies and Morris, 1993)), implicating low hepatic extraction in rodents and monkeys (below 30% of the hepatic blood flow), and medium hepatic extraction in dogs (in the range of 30%–70% of the hepatic blood flow). The volume of distribution at steady state (V_ss_) was determined to be 1.87, 1.68, 2.40, and 1.45 L/kg in mice, rats, dogs, and monkeys, respectively, corresponding to approximately 2.58-, 2.51-, 3.97-, and 2.09-fold greater than the corresponding volume of total body water (0.725, 0.668, 0.604, and 0.693 L/kg for mice, rats, dogs, and monkeys, respectively) (Davies and Morris, 1993), indicating partitioning into the peripheral tissue compartments. Following IV bolus administration, the plasma concentrations of amdizalisib decreased in a mono-exponential manner in rodents and dogs but showed a bi-exponential decline in monkeys, as evidenced by a terminal elimination half-life (T_1/2_) that was greater than the mean residence time (MRT) (Table 1).

After oral administration of amdizalisib, the exposure in terms of C_max_ and AUC generally increased dose-proportionally within the tested dose range (1–25 mg/kg) in rats. Similarly, at the oral dose levels of 0.5 to 25 mg/kg in dogs, the AUC_0-t_ of amdizalisib increased more than dose-proportionally and C_max_ increased almost dose-proportionally. The food effect on the PK of amdizalisib was observed in dogs. Compared with data in fasted dogs after a single oral dose of 25 mg/kg, food intake increased amdizalisib exposure, as indicated by the 2-fold increase in AUC_0-∞._ After PO dosing, absorption was rapid in rodents and monkeys (T_max_: 1.00–4.17 h) but absorbed slowly at 25 mg/kg in dogs (T_max_ 15.0 h). The absolute oral bioavailability (F_PO_%) of amdizalisib was calculated to be 101, 39.8, 43.1, and 30.8% in mice, rats, dogs, and monkeys, respectively, indicating a moderate to high oral bioavailability in preclinical species. The terminal T_1/2_ after oral dosing was longer than that after IV dosing in all species except monkeys, which may indicate multiple sites of absorption in rodents and dogs (Table 1).

### 3.2 Tissue distribution in SD rats

**FIGURE 2:**
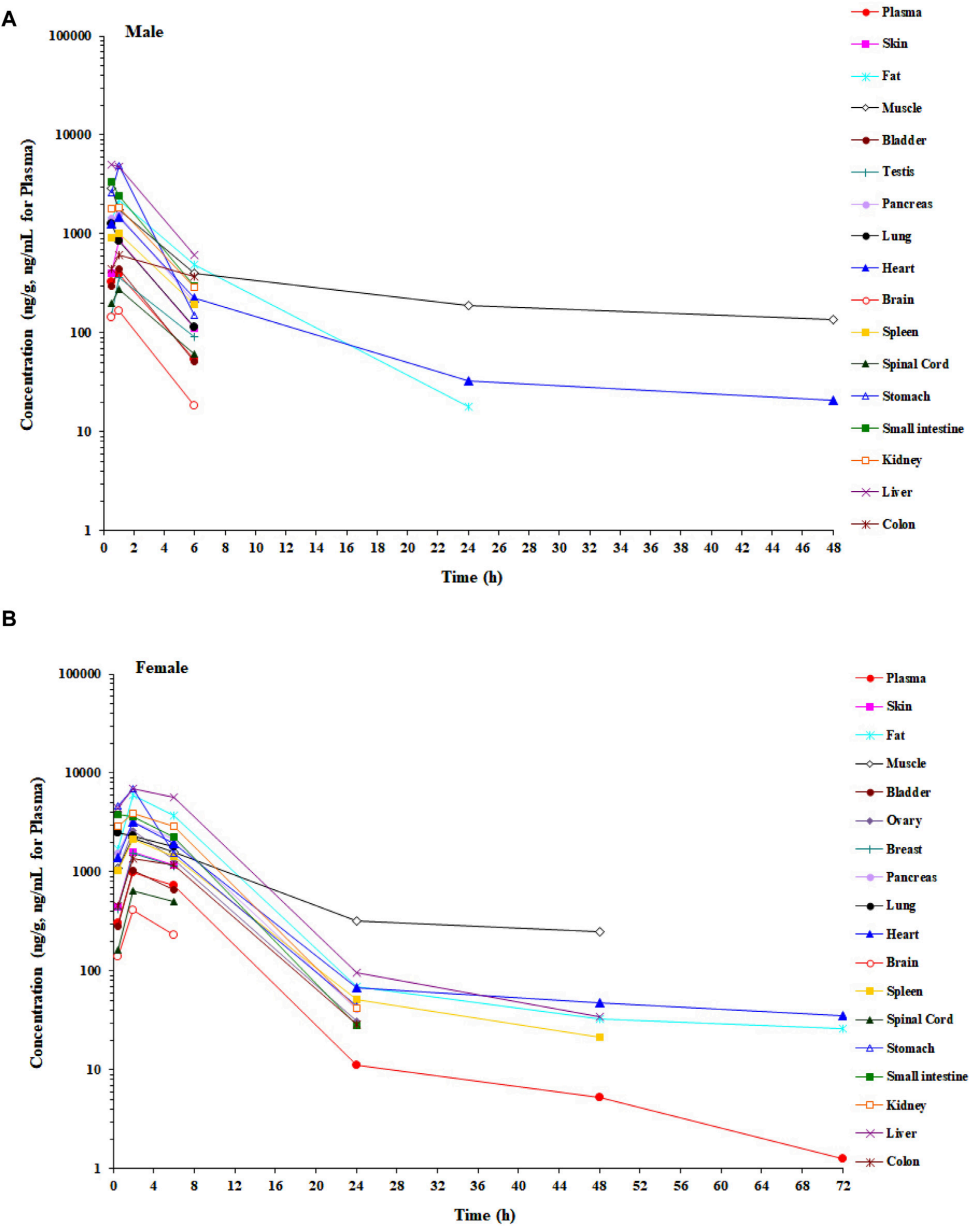
Mean concentration–time profiles of amdizalisib in male and female rat plasma and tissues after a single oral administration at 5 mg/kg (n = 3 or 6). **(A)** Male rat and **(B)** female rat.

In the tissue distribution study in SD rats, the concentrations of amdizalisib were determined in plasma and multiple tissues after a single oral administration of amdizalisib at 5 mg/kg. The tissue distribution profiles of amdizalisib at multiple time points and the main pharmacokinetic parameters in rats are shown in Figure 2; Supplementary Table S3, 4. Following a single oral dose of 5 mg/kg, the concentrations of amdizalisib attained their maximum at 0.5–2 h post-dose. The highest concentration was found in the liver (5,077 ng/g and 6,947 ng/g for male and female rats, respectively). Amdizalisib concentrations in most tissues were lower than the LLOQ (20 ng/g) at 24 h post-dose in male rats and 48 h post-dose in female rats, respectively. The elimination of amdizalisib in tissues was similar to that in plasma. The exposure (AUC_0-t_) in the liver was the highest among all tissues in both male and female rats, with about 12- and 8-fold higher than that in plasma in male and female rats, respectively. The tissues that had lower exposure than plasma were the testis, spinal cord, and brain in male rats and the skin, breast, bladder, spinal cord, and brain in female rats. The exposure (AUC) ratio of brain/plasma was 0.416 and 0.151 for male and female rats, respectively, indicating that amdizalisib is hard to pass through the blood–brain barrier.

### 3.3 Excretion/mass balance in SD rats

Following a single oral administration of 5 mg/100 μCi/kg [^14^C] amdizalisib, the excretion rate and amount of total cumulative radioactivity were similar between male and female BDI or BDC rats. As presented in Table 2, the recovery of radioactivity in mass balance studies was high (over 90%) in both BDI and BDC rats. The excretion of total radioactivity mainly occurred within 48 h in BDI rats post-dose, with over 80% of the total radioactivity recovered. The average total recovery of radioactivity at 168 h post-dose was 91.14% in BDI rats, in which 67.15% and 20.21% of the administered dose were recovered in feces and urine, respectively. The average total recovery of radioactivity in BDC rats was 91.15% of the administered dose within 72 h after the dose, in which 53.99% and 21.20% of the administered dose were recovered in the bile and urine, respectively, suggesting that at least 75.19% of the oral dose was absorbed. An average of 4.70%, 0.03%, and 29.80% of the administered dose of unchanged drug (the administered agent) was recovered in the bile, urine, and feces for female rats, respectively, and an average of 2.69%, <0.01%, and 23.20% of unchanged drug was recovered in the bile, urine, and feces for male rats, respectively.

**TABLE 2:** Cumulative recovery of [^14^C] amdizalisib-derived radioactivity from bile-duct intact (0–168 h) and bile-duct cannulation rat (0–72 h) after a single oral administration of 5 mg/100 μCi/kg.

Gender (n)	BDI rat				BDC rat				
	0–168 h				0–72 h				
	Urine (%)	Feces (%)	Cage washing (%)	Total recovery (%)	Urine (%)	Feces (%)	Bile (%)	Cage washing (%)	Total recovery (%)
Female (n = 3)	15.72 ± 2.62	71.15 ± 0.22	4.72 ± 2.41	91.59 ± 0.97	18.45 ± 4.01	19.81 ± 4.98	49.81 ± 2.07	2.24 ± 1.90	90.31 ± 0.91
Male (n = 3)	24.71 ± 4.46	63.14 ± 3.98	2.84 ± 0.17	90.69 ± 1.16	23.96 ± 8.70	8.98 ± 2.64	58.17 ± 4.68	0.89 ± 0.37	91.99 ± 1.88
Male and female (n = 6)	20.21 ± 5.91	67.15 ± 5.06	3.78 ± 1.84	91.14 ± 1.07	21.20 ± 6.77	14.40 ± 6.92	53.99 ± 5.61	1.57 ± 1.43	91.15 ± 1.61

Data expressed as the mean ± standard deviation.

### 3.4 Caco-2 transport

Amdizalisib, substrates, and control inhibitors were all soluble under the experimental conditions, and no significant non-specific adsorption of test compounds to the plastic wells was found at 37°C. The bidirectional transport permeability and the efflux ratio of amdizalisib across Caco-2 cell monolayers are shown in Table 3. Amdizalisib exhibited high permeability from the apical to the basolateral side (P_app, A-B_ around 40 × 10^−6^ cm/s), which was comparable with that of metoprolol (P_app, A-B_: 39.4 × 10^−6^ cm/s), a high permeability marker in this study. Amdizalisib showed low potential for efflux transport since ER were all below 2, indicating that amdizalisib may not be a substrate of the efflux transporters P-gp and/or BCRP. The transport of amdizalisib was linear within the tested concentrations ranging from 5 to 80 µM and over the time range from 30 to 150 min, as the P_app_ values almost remained constant.

**TABLE 3:** Bidirectional transport permeability and the efflux ratio of amdizalisib across Caco-2 cell monolayers.

Group	Substrate concentration (µM)	Incubation time (min)	P_app_ (×10–^6^ cm/s)		Efflux ratio
			A-B	B-A	
Bidirectional transport of control substrates and amdizalisib					
Atenolol	10	60	<0.431	0.32	>0.742
Metoprolol	10	60	39.4	36.8	0.935
Amdizalisib	5	60	38.5	50.4	1.31
	10	60	39.9	NA	NC
	20	60	40.9	52.3	1.28
	40	60	36.3	45.4	1.25
	60	60	42.2	47.9	1.13
	80	60	47.7	45.4	0.953
	10	30	45.7	69.5	1.52
	10	90	38.9	57.1	1.47
	10	120	36.9	56	1.52
	10	150	37.1	55.6	1.50
Inhibition of control inhibitors and amdizalisib on bidirectional transport of digoxin					
Digoxin	5	60	0.513	25.0	48.7
Digoxin + 10 µM Ko143	5	60	1.48	21.3	14.3
Digoxin + 1 µM LY335979	5	60	3.13	4.40	1.40
Digoxin + 0.1 µM amdizalisib	5	60	NA	22.7	NC
Digoxin + 0.5 µM amdizalisib	5	60	0.533	25.8	48.3
Digoxin + 2 µM amdizalisib	5	60	0.806	24.8	30.8
Digoxin + 5 µM amdizalisib	5	60	0.913	21.2	23.2
Digoxin + 10 µM amdizalisib	5	60	2.52	19.1	7.57
Digoxin + 20 µM amdizalisib	5	60	2.82	15.9	5.65
Digoxin + 40 µM amdizalisib	5	60	4.33	14.5	3.35
Digoxin + 60 µM amdizalisib	5	60	4.93	10.8	2.20
Digoxin + 80 µM amdizalisib	5	60	4.17	8.18	1.96
Inhibition of control inhibitors and amdizalisib on bidirectional transport of E3S					
E3S	2.5	60	2.79	77.8	27.9
E3S + 1 µM Ko143	2.5	60	10.1	8.35	0.828
E3S + 1 µM LY335979	2.5	60	3.96	87.9	22.2
E3S + 0.1 µM amdizalisib	2.5	60	3.06	56.7	18.5
E3S + 0.5 µM amdizalisib	2.5	60	2.78	52.2	18.7
E3S + 2 µM amdizalisib	2.5	60	6.40	65.4	10.2
E3S + 5 µM amdizalisib	2.5	60	3.36	60.2	17.9
E3S + 10 µM amdizalisib	2.5	60	3.85	46.2	12.0
E3S + 20 µM amdizalisib	2.5	60	NA	43.6	NC
E3S + 40 µM amdizalisib	2.5	60	4.82	40.2	8.34
E3S + 60 µM amdizalisib	2.5	60	6.19	31.4	5.07
E3S + 80 µM amdizalisib	2.5	60	5.70	41.7	7.31

NA, not applicable due to the leaky Caco-2 cell monolayers; NC, not calculated; A–B, apical side to basolateral side; B–A, basolateral side to apical side.

The inhibitory effects of amdizalisib on P-gp and BCRP activities were also investigated in Caco-2 cell monolayers. As displayed in Table 3, amdizalisib at concentrations of 0.5–5 µM showed no or slight inhibition on the transport of digoxin across the Caco-2 monolayer, while the extent of inhibition significantly increased when the concentration of amdizalisib was raised to 10 µM and above. The percentage of inhibition on P-gp and BCRP by control inhibitors and amdizalisib is shown in Supplementary Table S5. When the percentage of inhibition (Y) was plotted against the logarithmic concentration of amdizalisib (X), they were well fitted with the sigmoidal dose–response curve (adjusted R^2^ = 0.982) using software Origin Graph 8.6 (Northampton, MA, United States). Amdizalisib inhibited digoxin transport with an estimated IC_50_ of 22.9 µM. Amdizalisib at concentrations of 0.1–5 µM slightly inhibited the transport of E3S across the Caco-2 monolayer, and the extent of inhibition showed no obvious increase when the concentration of amdizalisib further increased from 10 µM to 80 μM, with the maximum inhibition not exceeding 66.4% up to 80 µM of amdizalisib.

### 3.5 Plasma protein binding and whole blood/plasma ratio

The plasma protein binding (PPB) data of amdizalisib are listed in Table 4. In all species examined, the PPB rate of amdizalisib showed a constant value of approximately 90% at the concentration range of 0.1–20 μM, suggesting that when considering the interspecies allometric scaling of PK parameters, the correction with plasma unbound fraction (f_up_) may not be necessary. Recovery and stability of amdizalisib were assessed post-incubation, and both were >90% in all cases (data not shown), indicating the PPB data was reliable.

**TABLE 4:** Binding fractions of amdizalisib to plasma proteins in different species.

	Concentration (µM)	Mouse (%)	Rat (%)	Dog (%)	Monkey (%)	Human (%)
Amdizalisib	0.1	91.1 ± 0.95	87.2 ± 1.72	87.7 ± 5.15	90.6 ± 4.56	94.0 ± 0.86
	1	91.5 ± 1.01	90.2 ± 0.65	89.1 ± 0.83	89.7 ± 0.22	92.8 ± 0.54
	20	91.3 ± 0.89	89.9 ± 0.37	88.8 ± 0.69	88.9 ± 0.38	92.4 ± 0.15

Data expressed as the mean ± standard deviation.

The blood-to-plasma partitioning data of amdizalisib are shown in Table 5. The blood-to-plasma ratio (R_B_) of amdizalisib at 0, 10, and 60 min was 0.615, 0.680, and 0.797 in human whole blood, 1.01, 0.874, and 0.968 in dog blood, and 1.08, 1.16, and 1.03 in rat blood, respectively. There was a slight increase in the R_B_ from 0 to 60 min in human whole blood, while no volatility of the R_B_ was observed in rat and dog whole blood, indicating that the partition equilibrium rate of amdizalisib between RBC and plasma was fast in rat and dog whole blood and slow in human whole blood. Combining the unbound fraction (f_up_) of amdizalisib in the plasma of rats, dogs, and humans at a concentration of 1 μM, the unbound fractions of amdizalisib in whole blood were estimated to be similar (about 10%) across the species tested.

**TABLE 5:** Blood-to-plasma partition ratio (R_B_) and the unbound fractions (f_uB_) of amdizalisib in the blood of rats, dogs, and humans.

Species	Time (min)	f_up_	R_B_	f_uB__(%)_
Rat	0	0.0980	1.08	9.11
	10		1.16	8.41
	60		1.03	9.49
Dog	0	0.109	1.01	10.9
	10		0.874	12.5
	60		0.968	11.3
Human	0	0.0720	0.615	11.7
	10		0.680	10.6
	60		0.797	9.02

### 3.6 *In vitro* metabolic stability in liver microsomes

As shown in Table 6, amdizalisib was most stable in male human liver microsomes, with the parent drug remaining larger than 80% after incubation for 120 min. The *in vitro* t_1/2_ of amdizalisib was 62.4, 96.3, 37.9, and 108 min in the liver microsomes of male monkeys, dogs, rats, and mice, respectively. CL_sys_ was calculated to be 17.8, 12.4, 30.0, and 32.3 mL/min/kg, respectively. In the liver microsomes of female humans, monkeys, dogs, rats, and mice, the *in vitro* t_1/2_ of amdizalisib was 139, 97.6, 41.5, 187, and 102 min, respectively, and CL_sys_ was calculated to be 4.52, 13.3, 18.8, 10.7, and 33.6 mL/min/kg, respectively. According to the predicted systemic clearance (CL_sys_), amdizalisib showed low clearances in human and female rat liver microsomes (<30% hepatic blood flow), medium clearances in monkey, dog, and mouse liver microsomes (<70% but >30% hepatic blood flow), and high clearance in male rat liver microsomes (>70% hepatic blood flow). The gender difference in predicted CL_sys_ was only observed in rats. The predicted CL_sys_ is generally well consistent with the CL_sys_ obtained from *in vivo* studies across all preclinical species, with differences within two- to three-fold of the measured clearance. A gender difference was also observed in CL_sys_ obtained from the *in vivo* rat PK study. FMO showed no contribution to amdizalisib metabolism in liver microsomes in all species.

**TABLE 6:** Liver microsomal stability and scaled hepatic CL across species for amdizalisib.

Species	FMO contribution	*in vitro* t_1/2_	Predicted CL_*int, iv vivo*_	Predicted CL_*sys*_	*in vitro* t_1/2_	Predicted CL_*int, iv vivo*_	Predicted CL_*sys*_
		(min)	(mL/min/kg)	(mL/min/kg)	(min)	(mL/min/kg)	(mL/min/kg)
		Male			Female		
Human	Normal	S*	NA	NA	139	5.78	4.52
	FMO deactivation	S*	NA	NA	119	6.71	5.07
Monkey	Normal	62.4	30.0	17.8	97.6	19.2	13.3
	FMO deactivation	74.5	25.1	15.9	103	18.1	12.8
Dog	Normal	96.3	20.7	12.4	41.5	48.1	18.8
	FMO deactivation	100	19.9	12.1	39.6	50.4	19.2
Rat	Normal	37.9	65.9	30.0	187	13.3	10.7
	FMO deactivation	41.7	59.8	28.7	169	14.8	11.6
Mouse	Normal	108	50.4	32.3	102	53.6	33.6
	FMO deactivation	114	48.0	31.3	103	52.8	33.3

CL_*int, iv vivo*_, *in vivo* intrinsic clearance; CL_*sys*_, systemic clearance; FMO, flavin monooxygenase; NA, not available. *: Amdizalisib was stable in the liver microsomes (Parent drug remaining >80% after incubation for 120 min).

### 3.7 CYP inhibition

Without pre-incubation (reversible CYP inhibition), amdizalisib inhibited CYP2C8 and CYP2C9 activities with IC_50_ values of 30.4 and 10.7 μM, respectively. Amdizalisib did not significantly inhibit CYP1A2, CYP3A4/5, CYP2B6, CYP2C19, CYP2D6, and CYP2E1 activities with IC_50_ greater than 50 µM. The inhibition effects of amdizalisib on activities of CYP450 isoforms are provided in Supplementary Table S6. Positive inhibitors showed inhibition of the activity of each CYP450 isoform with consistent IC_50_ values for in-house studies (data not shown). In addition, amdizalisib exhibited no significant inhibition on CYP1A2, CYP2B6, CYP2C8, CYP2C19, and CYP2D6, with less than 10% activity loss following 30 min of pre-incubation with NADPH. Amdizalisib caused more than 10% but less than 20% activity loss of CYP2C9 (14.6%), CYP3A4/5 (substrate: testosterone, 11.7%), and CYP3A4/5 (substrate: midazolam, 16.9%), indicating weak time-dependent inactivation of these CYP isoforms. All positive inhibitors showed potent time-dependent inhibition of the relevant target CYP isoforms (data not shown).

### 3.8 CYP induction

At the concentrations tested, amdizalisib did not show an obvious cytotoxic effect on cell viability except that amdizalisib at 20 μM exhibited cytotoxicity against human hepatocytes in one donor (data not shown) in the evaluation of CYP2C induction. As shown in Supplementary Table S7, 8, based on the enzyme activity, amdizalisib at up to 30.0 µM was not considered an inducer for CYP1A2, CYP2B6, and CYP3A4 in all three donors, and at concentrations of up to 20 µM, it showed no induction potential on CYP2C8, CYP2C9, and CYP2C19 enzyme activities in all three donors. The results from gene expression levels indicate that amdizalisib at concentrations of 10.0–30.0 μM exhibited induction effects on CYP1A2 and CYP2B6 in two donors (BXW and NFX), with induction folds of 2.57–3.88 and 2.50–6.14 compared to the vehicle control, respectively. BXW showed the induction of CYP1A2 expression with E_max_ of 3.88-fold and EC_50_ of 9.57 μM, and the induction of CYP2B6 expression with E_max_ of 5.42-fold and EC_50_ of 5.73 μM. NFX showed the induction of CYP1A2 expression with E_max_ of 3.16-fold and EC_50_ of 7.73 μM and the induction of CYP2B6 expression with E_max_ of 3.39-fold and EC_50_ of 4.48 μM. In one donor (XSM), amdizalisib was considered an inducer for CYP1A2 at concentrations of 10.0 μM and 20.0 μM (2.49- and 2.06-fold, respectively). In the same donor, amdizalisib also induced CYP2B6 at concentrations ranging from 3.00 to 30.0 μM, with an induction fold of 2.19–8.96 compared to the vehicle control. XSM showed the induction of CYP1A2 expression with E_max_ of 2.17-fold and EC_50_ of 5.50 μM and the induction of CYP2B6 expression with E_max_ of 7.94-fold and EC_50_ of 3.38 μM. The induction of CYP3A4 was observed at concentrations of 1.00 and 3.00 μM (2.59- and 3.52-fold, respectively) in hepatocytes from one of the three donors (XSM) and at concentrations ranging from 10.0 to 30.0 μM in hepatocytes from all three donors (3.59- to 35.5-fold). BXW, XSM, and NFX showed the induction of CYP3A4 expression with E_max_ of 6.97-, 35.7-, and >26.5-fold and with EC_50_ of 6.11, 9.91, and >30.0 μM, respectively. Amdizalisib showed greater than 2-fold induction on CYP2C9 mRNA expression in all three donors (2.91- to 3.37-fold). Amdizalisib at a concentration of up to 20 µM showed no induction potential on CYP2C8 mRNA expression. All positive controls showed induction potential for corresponding CYPs, and flumazenil (negative control) did not induce CYP2C enzyme activity and gene expression levels. The stability of amdizalisib following the last incubation in human hepatocytes is provided in Supplementary Table S9, 10.

### 3.9 Transporter inhibition

Amdizalisib was found to markedly inhibit substrate uptake in OAT1-, OATP1B1-, OATP1B3-, and MATE1-over-expressing HEK293 cells by more than 50% at the concentration ranging from 10.0 to 100 μM (detailed calculation in Supplementary Table S11). The inhibitory potency of amdizalisib on the specific substrate uptake mediated by SLC transporters was evaluated by the IC_50_ values derived from the dose–response curves (Supplementary Figure S1). As shown in Table 7, the calculated IC_50_ values of amdizalisib on OAT1, OAT3, OATP1B1, OATP1B3, MATE1, and MATE2-K was 4.14, 41.1, 7.27, 5.03, 16.2, and 38.2 μM, respectively. Amdizalisib had mild or no effect on the OCT2-mediated metformin uptake with IC_50_ value > 100 μM (percentage inhibition less than 50%). 100% inhibition was achieved in all positive controls (Supplementary Table S11).

**TABLE 7:** Inhibition of substrate uptake into transporter-transfected HEK293 cells by amdizalisib.

Transporter	Probe substrate	Compound	IC_50_ (μM)
OAT1	4-aminohippuric acid	Amdizalisib	4.14
		Probenecid	5.80
OAT3	Estrone 3-sulfate	Amdizalisib	41.1
		Probenecid	14.5
OCT2	Metformin	Amdizalisib	>100
		Verapamil	10.1
OATP1B1	β-estradiol 17-(β-D-glucuronide)	Amdizalisib	7.27
		Rifampicin	0.866
OATP1B3	β-estradiol 17-(β-D-glucuronide)	Amdizalisib	5.03
		Rifampicin	0.611
MATE1	Tetraethylammonium	Amdizalisib	16.2
		Pyrimethamine	0.0125
MATE2-K	Tetraethylammonium	Amdizalisib	38.2
		Pyrimethamine	0.0136

### 3.10 Human PK projection

**FIGURE 3:**
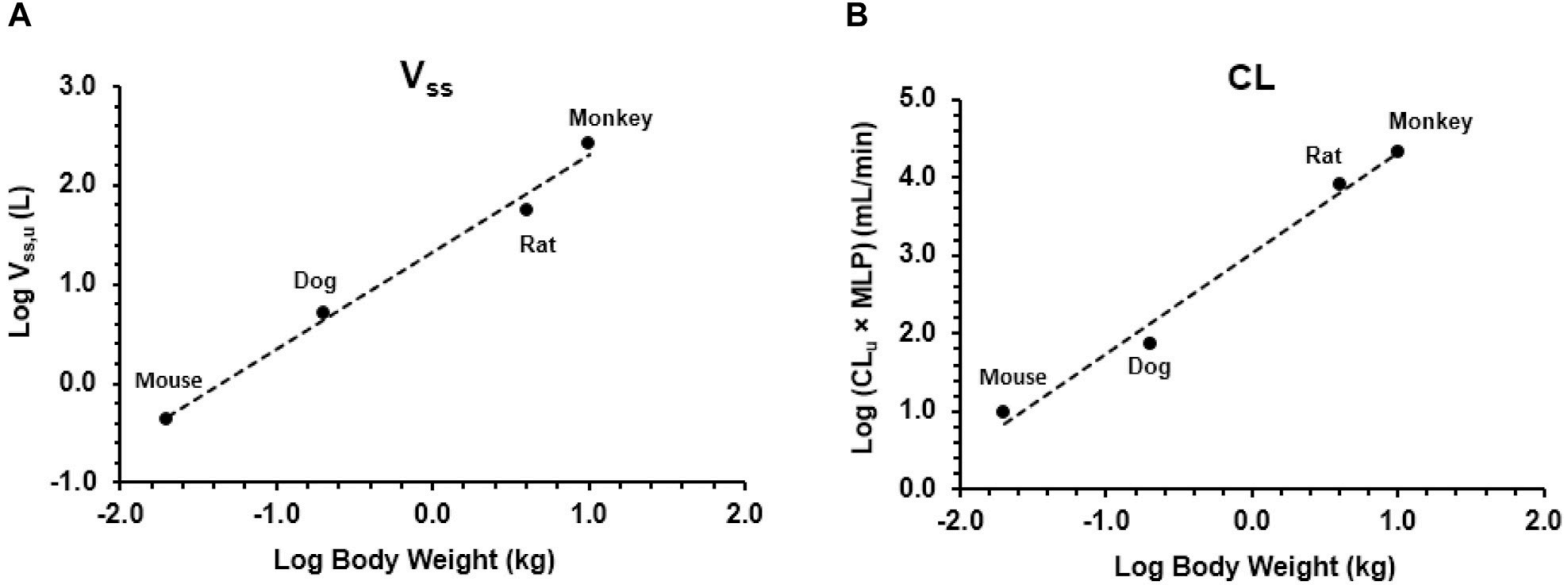
Allometric scaling of plasma clearance and volume of distribution at steady state using pharmacokinetic data from mice, rats, dogs, and monkeys. Data presented as individual values. **(A)** Volume of distribution and **(B)** clearance.

As shown in Figure 3, V_ss,u_ and CL_u_ × MLP showed good correlation with BW with corresponding correlation coefficients of 0.9899 and 0.9874, respectively. The application of allometric scaling with the correction of MLP and f_u_ resulted in a predicted CL value of 2.57 mL/min/kg, and thus, CL/F was calculated to be 4.78 mL/min/kg (assuming F% = 53.7%, an average of F% from all preclinical species), which was close to the reported human CL_ss_/F of 3.25 mL/min/kg (within two-fold error). Predicted human V_ss,u_ was 1.35 L/kg, and thus, V_ss_/F was calculated to be 2.51 L/kg, showing good agreement with the observed human value of 2.33 L/kg.

## 4 Discussion

Amdizalisib is an orally available, highly selective, and potential best-in-class small-molecule PI3Kδ inhibitor with high potency. Amdizalisib is now being developed at the clinical stage for the treatment of patients with FL and MZL. Extensive characterization of the preclinical pharmacokinetics of a potential drug candidate has a great influence on the development process. In this paper, the *in vitro* and *in vivo* PK properties of amdizalisib were evaluated, including *in vivo* PK studies in multiple preclinical species, metabolic stability, permeability, protein binding, whole blood/plasma ratio, CYP inhibition, CYP induction, and transporter inhibition, and its potential human PK properties were also assessed.

The oral absorption of amdizalisib appears to be relatively fast, with a T_max_ of 1 h to 4.17 h in rodents and monkeys, while T_max_ increased from 1.92 to 15.0 h when the oral dose increased from 0.5 to 25 mg/kg in dogs, suggesting that higher doses can result in slower absorption for amdizalisib. Amdizalisib showed good oral bioavailability (F > 30%) in preclinical species, which is in line with its physicochemical properties, such as tPSA <120Å^2^ and MW < 500 Da (Van de Waterbeemd, 2003), and also in accordance with its high permeability across Caco-2 cell monolayers and good metabolic stability. The mice showed much higher oral bioavailability of amdizalisib than other species, which is likely due to the different drug dissolution behavior of amdizalisib in the GI tract between species (Tanaka et al., 2013). Since amdizalisib is a lipophilic compound with poor water solubility (Table 8) and high permeability, and no significant difference in systemic clearance was observed between preclinical species, it is assumed that the dissolution rate of amdizalisib in the GI tract could be the rate-limiting step for its oral absorption. Therefore, the species-difference in factors (e.g., bile acid and phospholipid concentrations) that can influence the dissolution rate of drugs may cause the difference in bioavailability observed between mice and other species. The significant food effect on the oral absorption of amdizalisib was observed in dogs, as evidenced by the increased plasma exposure (AUC_0-inf_ and C_max_) and faster absorption (shorter time to achieve C_max_) under fed status than under fasted conditions. However, in the clinical food effect study of amdizalisib after the single oral dose at 30 mg conducted in Chinese healthy volunteers (Cao et al., 2023), the result showed that the high-fat diet can reduce the oral absorption rate of amdizalisib (median T_max_ prolonged from 1 h to 4 h and the geometric mean of C_max_ reduced by 36% after food intake), and no significant effect on the extent of oral absorption (AUC) was observed. This complete difference observed between dogs and humans demonstrated that the food effect study in dogs cannot be translated to humans simply due to the difference in gastric emptying and small intestine transit time, as well as the variable intestinal pH under the fed and fasted states between dogs and humans (Dressman, J. B., 1986).

**TABLE 8:** ADME properties of amdizalisib.

Amdizalisib	
ADME properties	Data
Solubility	0.025 mg/mL at pH 7.78
*In vivo* PK	CL = 14.7 (mice), 9.12 (rats), 11.8 (dogs), 11.3 (monkeys) mL/min/kg
Caco-2	P_app_ A–B = 47.7; B–A = 45.4 (×10–^6^ cm/s), efflux ratio = 0.953 at 80 µM
Transporter substrate	Not a substrate of P-gp and BCRP
Plasma protein binding	Approximately 90% in all species
Blood-to-plasma ratio	R_B_ = 1.03 (rats), 0.968 (dogs), 0.797 (humans)
CYP inhibition	IC_50_ = 30.4 μM (CYP2C8), 10.7 μM (CYP2C9), >50 µM (CYP1A2, 3A4/5, 2B6, 2C19, 2D6, 2E1)
CYP induction	>2-fold induction on CYP1A2, 2B6, 3A4, 2C9 mRNA expression at 20 uM
Transporter inhibition	IC_50_ = 22.9 µM (P-gp), 4.14 µM (OAT1), 41.1 µM (OAT3), >100 µM (OCT2), 7.27 µM (OATP1B1), 5.03 µM (OATP1B3), 16.2 µM (MATE1), 38.2 µM (MATE2-K)

V_ss_ of amdizalisib was similar across different preclinical species (mice, rats, dogs, and monkeys), with values of 2- to 4-fold greater than the total body water volume, which suggests extensive extravascular tissue distribution. This is further supported by the study results of tissue distribution in SD rats, in which amdizalisib was found to be distributed to various tissues within 1–2 h and with tissue-to-plasma concentration ratios higher than 1 in most tissues following oral administration. Among them, the liver showed the highest exposure with a tissue-to-plasma concentration ratio higher than 10, and the exposure of amdizalisib in the intestines was only approximately 3- to 6-fold higher than in plasma, which is much lower than other PI3Kδ inhibitors such as idelalisib (50-fold higher) (FDA, 2014), duvelisib (100-fold higher) (FDA, 2017), copanlisib (190-fold higher) (FDA, 2018), and umbralisib (100-fold higher) (FDA, 2021). It is well-known that P-gp is widely expressed on the intestinal epithelium as an efflux transporter; it can not only prevent oral drug intestinal absorption but can also contribute to the elimination of many drugs by mediating their direct secretion from the blood into the intestinal lumen (Van Asperen et al., 1998), which will also possibly lead to the specific intestinal distribution of drugs. With the exception of umbralisib, all the above-mentioned marketed PI3Kδ inhibitors, including idelalisib, duvelisib, and copanlisib, were P-gp substrates, while umbralisib showed the highest V_z_/F among these drugs in human (FDA, 2014; FDA, 2017; FDA, 2018; FDA, 2021). Amdizalisib showed low V_z_/F in humans and was not a substrate of P-gp. Both of these two characteristics determine the limited exposure of amdizalisib to the intestinal wall, which will be helpful in reducing its GI side effects. This assumption was supported by the reported GI toxicity in the clinical trials. As reported, ≥Grade 3 GI toxicity, including diarrhea and colitis, is one of the most common adverse effects for idelalisib, duvelisib, copanlisib, and umbralisib, with incidences all higher than 10% (FDA, 2014; FDA, 2017; FDA, 2018; FDA, 2021). However, for amdizalisib, less than 5% of serious GI toxicity was observed in humans, with Grade 3 diarrhea being low (2.2%), and no colitis cases were reported at the recommended phase 2 dose (RP2D: 30 mg) in a phase Ib study (n = 90) (European Society for Medical Oncology, 2021).

The prediction of plasma clearance from female human microsomal data produced a value of 4.52 mL/min/kg, and allometric scaling (correction with MLP) of *in vivo* clearance data from all four preclinical species generated a value of 2.57 mL/min/kg (CL/F = 4.78 mL/min/kg assuming F% = 53.7%); both predicted clearances would characterize amdizalisib as a low clearance compound (<30% of the liver blood flow) in humans, which is found to be in good agreement with the clearance observed in oncology patients.

The excretion study reveals that the recovery of administered radioactivity was high (over 90% of the dose) after oral administration of [^14^C] amdizalisib to rats, with 54% and 21% of the dose being excreted into bile and urine, respectively, indicating the importance of urinary and biliary excretion of amdizalisib. The excretion of the amdizalisib prototype was found to be low (<30%) in feces, bile, and urine, suggesting that the absorbed amdizalisib is mainly excreted via bile and urine as metabolites.

The *in vitro* experiments indicated that amdizalisib was not the substrate of P-gp and BCRP and showed inhibition potential on P-gp but not on BCRP. Combining the *in vitro* determined IC_50_ and calculated I_gut_ (intestinal luminal concentration of the interacting drug calculated as the RP2D dose/250 mL) (FDA, 2020), it is predicted that the inhibition potential of amdizalisib on P-gp (I_gut_/IC_50_ ≥ 10) may cause the increased exposure of P-gp substrates following the co-administration of amdizalisib and P-gp substrates. The impact of amdizalisib as an inhibitor and inducer of P450 enzymes was evaluated using human liver microsomes and human hepatocytes, respectively. Studies with HLMs have highlighted that amdizalisib has the potential to act as a weak inhibitor of CYP2C8 and CYP2C9 (IC_50_: 30.4 and 10.7 μM), and no inhibition was observed on the rest of the major P450 enzymes (CYP1A2, CYP3A4/5, CYP2B6, CYP2C19, CYP2D6, and CYP2E1, IC_50_ > 50 μM), while at the maximal unbound plasma concentration of amdizalisib at steady state (∼52.1 nM), inhibition on CYP2C8 and CYP2C9 by amdizalisib at 30 mg QD in humans is unlikely (FDA, 2020). No time-dependent inhibition of P450 enzymes was observed for amdizalisib, suggesting that the formation of reactive metabolites is unlikely. Therefore, the risk of serious drug–drug interactions caused by competitive or time-based inhibition of P450 enzymes is considered to be low for amdizalisib. Although an *in vitro* study showed that amdizalisib had the potential to induce CYP1A2, CYP2B6, CYP3A4, and CYP2C9, only CYP2B6 and CYP3A4 may possibly be induced by amdizalisib according to the equation from the basic kinetic model (R_3_ value ≤0.8) that is used to predict induction potential *in vivo* (FDA, 2020). Studies with SLC transporter-overexpressing HEK293 cells indicated that amdizalisib inhibited the function of human OATP1B1 and OATP1B3 with IC_50_ values of 7.27 and 5.03, respectively, and the calculated R value was >1.1 (3.8 for OATP1B1 and 5.1 for OATP1B3, respectively) according to the equation recommended by the US FDA Guidance (FDA, 2020), suggesting that clinically significant DDI should be considered. The potential impact of amdizalisib on the activities of renal OATs, OCT2, MATE-1, and MATE2-K was low (calculated R value < 1.1), indicating that amdizalisib has a low risk of affecting renal elimination of concomitantly administered substrate drugs.

## 5 Conclusion

The preclinical data gathered in this work provided evidence that amdizalisib exhibited good oral bioavailability with low clearance and extensive tissue distribution in preclinical species (Table 8). Amdizalisib showed high cell permeability without efflux transporter substrate liability, adequate metabolic stability, and a low potential to cause drug–drug interactions. *In vitro* scaling of liver microsomal clearance data showed good agreement with *in vivo* clearance. After oral administration, amdizalisib was mainly eliminated via bile and urine as metabolites. Amdizalisib showed much lower exposure in the intestinal wall in rats, possibly related to its lower GI toxicity observed in humans compared to other PI3Kδ inhibitors. The excellent receptor selectivity combined with its unique PK properties makes it a valuable compound in this class for further development.

## Data Availability

The original contributions presented in the study are included in the article/Supplementary Material; further inquiries can be directed to the corresponding author.

## Abbreviations

ADME, absorption, distribution, metabolism, excretion; AUC_0-t_, area under the plasma concentration–time curve from time zero to the last quantifiable time point; AUC_0-inf_, area under the plasma concentration–time curve from time zero to infinity; BW, body weight; BDC, bile duct cannulated; C_max_, maximum plasma concentration; CLss, clearance at steady state; CLint, intrinsic clearance; CLsys, systemic clearance; CYP, cytochrome P450; DDI, drug–drug interaction; FPO, oral bioavailability; FDA, Food and Drug Administration; GI, gastrointestinal; IV, intravenous; IC_50_, concentration achieving 50% inhibition; MLP, maximum lifespan potential; PO, per os; PI3K, phosphatidylinositol 3-kinase; PK, pharmacokinetics; RP2D, recommended phase 2 dose; RoE, rule of exponents; T_max_, time corresponding to C_max_; t_1/2_, half-lives; tPSA, topological polar surface area; Vss, volume of distribution at steady state.
