# Supplementary material for: Preclinical pharmacokinetic characterization of amdizalisib, a novel PI3Kδ inhibitor for the treatment of hematological malignancies
Source: Front Pharmacol. 2024 Jun 14;15:1392209. doi: 10.3389/fphar.2024.1392209 (PMC11211886; doi:10.3389/fphar.2024.1392209)
Supplement: Supplementary file 1 [file DataSheet1.docx]

1. **Data analysis of CYP induction by amdizalisib**

The fold of vehicle control and the percent of positive control were calculated with the formulas below:

Fold of vehicle control = CYPs activity of test compound (or positive control) treated sample / CYPs activity of vehicle control treated sample

$$\text{\% of positive control = [(fold change in treated sample)-1] / [(fold change in positive control)-1] × 100}$$

Based on the enzyme activity, a minimum of 2-fold increase compared to vehicle control for the positive control and ≥40% of the positive control for the test article was considered as the cutoff for a positive signal in *in vitro* induction assay.

To account for variation in RNA yields and reverse transcription polymerase chain reaction (RT-PCR), the gene of interest in all samples was normalized to an internal control gene (18S ribosomal RNA) (Ct_target gene_ – Ct_18S_= ΔCt). The relative quantitation or change in mRNA level of selected genes induced by each test compound was expressed in relation to the vehicle control sample (ΔCt_compound_ –ΔCt_vehicle_= ΔΔCt). Fold changes in gene expression were determined by taking 2 to the power of this value (2^-ΔΔCt^). The percent of positive control was calculated with the formula below:

$$\text{\% of positive control = [(fold change in treated sample)-1] / [(fold change in positive control)-1] × 100}$$

Based on the gene expression, a minimum of 4-fold increase of positive control compared to vehicle control, a minimum of 2-fold increase of the test article compared vehicle control and increased in a concentration-dependent manner was considered as the cutoff for a positive signal in *in vitro* induction assay.

1. **Bioanalytical method of amdizalisib**

All the final solution samples were analyzed to determine the concentrations of amdizalisib by a liquid chromatography coupled with tandem mass spectrometry (LC-MS/MS). ESI source and multiple-reaction monitoring (MRM) mode of *m/z* 391.1 to 256.1 was used for detection of amdizalisib with the optimal ion source and MS parameters. The universal and validated analytical methods were shown in Table 1 and Table 2, respectively.

**Table 1S** Universal chromatographic method.

| Mobile phase | A: Water with 0.1% formic acid (FA)  B: Acetonitrile with 0.1% formic acid (FA) | | |
| --- | --- | --- | --- |
| Column Oven: | 30 ^o^C | | |
| Injection volume: | 5 μL | | |
| Acquisition time: | 3.5 min | | |
| Autosampler temperature | Approx. 8 ^o^C | | |
| Autosampler loop size: | 20 μL | | |
| Autosampler needle wash: | Wash 1: Methanol: Acetonitrile: Isopropyl alcohol: Ethyl acetate: Water = 1: 1: 1: 1: 1, containing 0.1% FA  Wash 2: Methanol: Acetonitrile: Water = 1: 1: 2, containing 0.1% FA | | |
| Time | Flow (µL/min) | Mobile phase A | Mobile phase B |
| 0 min | 500 | 95% | 5% |
| 2.00 min | 500 | 0% | 100% |
| 2.50 min | 500 | 0% | 100% |
| 2.51 min | 500 | 95% | 5% |
| 3.50 min | 500 | 95% | 5% |

**Table 2S** Validated chromatographic method.

| Mobile phase | A: Water with 0.025% formic acid & 2 mM Ammonium acetate  B: Methanol with 0.025% formic acid & 2 mM Ammonium acetate | | |
| --- | --- | --- | --- |
| Column Oven: | 30 ^o^C | | |
| Injection volume: | 1 μL | | |
| Acquisition time: | 3.2 min | | |
| Autosampler temperature | Approx. 8 ^o^C | | |
| Autosampler needle wash: | Methanol: Water =1: 1 | | |
| Time | Flow (µL/min) | Mobile phase A | Mobile phase B |
| 0 min | 600 | 50% | 50% |
| 0.50 min | 600 | 40% | 60% |
| 1.50 min | 600 | 5% | 95% |
| 2.20 min | 600 | 5% | 95% |
| 2.30 min | 600 | 50% | 50% |
| 3.20 min | 600 | 50% | 50% |

1. **The main pharmacokinetic parameters of amdizalisib in rat tissues**

**Table 3S** Main pharmacokinetic parameters of amdizalisib after a single oral administration at 5 mg/kg in male SD rats (n=3).

| Tissue | Parameters | | | | |
| --- | --- | --- | --- | --- | --- |
|  | AUC_0-t_ | C_max_ | T_max_ | t_1/2_ | AUC_0-t_ ratio of tissue/plasma |
|  | h·ng/g for tissue  h·ng/mL for plasma | ng/g for tissue  ng/mL for plasma | h | h |  |
| Liver | 17191^&^ | 5077 | 0.500 | 1.76 | 12.4 |
| Muscle | 16564^ | 2896 | 0.500 | 27.7 | 12.0 |
| Stomach | 15320^&^ | 4953 | 1.00 | - | 11.1 |
| Fat | 12483* | 2210 | 1.00 | 3.42 | 9.02 |
| Small intestine | 9064^&^ | 3377 | 0.500 | 1.60 | 6.55 |
| Heart | 8180^ | 1469 | 1.00 | 8.52 | 5.91 |
| Kidney | 6639^&^ | 1823 | 1.00 | - | 4.80 |
| Pancreas | 5497^&^ | 1553 | 1.00 | - | 3.97 |
| Spleen | 3718^&^ | 1008 | 1.00 | - | 2.69 |
| Lung | 3257^&^ | 1264 | 0.500 | 1.65 | 2.35 |
| Skin | 2858^&^ | 863 | 1.00 | - | 2.07 |
| Colon | 2814^&^ | 608 | 1.00 | - | 2.03 |
| Bladder | 1499^&^ | 444 | 1.00 | - | 1.08 |
| Plasma | 1383^&^ | 394 | 1.00 | - | 1.00 |
| Testis | 1312^&^ | 366 | 1.00 | - | 0.948 |
| Spinal Cord | 1013^&^ | 277 | 1.00 | - | 0.732 |
| Brain | 575^&^ | 166 | 1.00 | - | 0.416 |

Dash (-) indicates not determined; ^&^AUC_0-6h_; *AUC_0-24h_; ^AUC_0-48h._

**Table 4S** Main pharmacokinetic parameters of amdizalisib after a single oral administration at 5 mg/kg in female SD rats (n=3).

| Tissue | Parameters | | | | |
| --- | --- | --- | --- | --- | --- |
|  | AUC_0-t_ | C_max_ | T_max_ | t_1/2_ | AUC_0-t_ ratio of tissue/plasma |
|  | h·ng/g for tissue  h·ng/mL for plasma | ng/g for tissue  ng/mL for plasma | h | h |  |
| Liver | 89024^ | 6947 | 2.00 | 5.61 | 7.82 |
| Fat | 61766^#^ | 6030 | 2.00 | 33.9 | 5.43 |
| Kidney | 45718* | 3950 | 2.00 | 3.22 | 4.02 |
| Stomach | 41360* | 6957 | 2.00 | 3.15 | 3.63 |
| Small intestine | 39168* | 3860 | 0.500 | 3.04 | 3.44 |
| Pancreas | 34420* | 3227 | 2.00 | 3.38 | 3.02 |
| Heart | 34373^#^ | 3227 | 2.00 | 49.9 | 3.02 |
| Muscle | 34333^ | 2203 | 2.00 | 14.4 | 3.02 |
| Spleen | 24041^ | 2157 | 2.00 | 6.62 | 2.11 |
| Ovary | 23191* | 2623 | 2.00 | 3.40 | 2.04 |
| Colon | 17267* | 1360 | 2.00 | 3.77 | 1.52 |
| Lung | 12468^&^ | 2490 | 0.500 | 11.6 | 1.10 |
| Plasma | 11385^#^ | 993 | 2.00 | 15.3 | 1.00 |
| Skin | 7121^&^ | 1580 | 2.00 | - | 0.625 |
| Breast | 6935^&^ | 1540 | 2.00 | - | 0.609 |
| Bladder | 4448^&^ | 1027 | 2.00 | - | 0.391 |
| Spinal Cord | 2917^&^ | 638 | 2.00 | - | 0.256 |
| Brain | 1723^&^ | 410 | 2.00 | - | 0.151 |

Dash (-) indicates not determined; ^&^AUC_0-6h_; *AUC_0-24h_; ^AUC_0-48h_; ^#^AUC_0-72h._

1. **The percentage of inhibition on P-gp and BCRP by control inhibitors and amdizalisib**

**Table 5S** The calculated percentage of inhibition on P-gp and BCRP by control inhibitors and amdizalisib.

| Co-incubation inhibitor or amdizalisib and concentration | Substrate | Percentage of inhibition (%) |
| --- | --- | --- |
| P-gp inhibition | | |
| 10 µM Ko143 | Digoxin | 19.0 |
| 1 µM LY335979 | Digoxin | 94.8 |
| 0.5 µM Amdizalisib | Digoxin | -3.22 |
| 2 µM Amdizalisib | Digoxin | 1.75 |
| 5 µM Amdizalisib | Digoxin | 17.0 |
| 10 µM Amdizalisib | Digoxin | 32.3 |
| 20 µM Amdizalisib | Digoxin | 46.5 |
| 40 µM Amdizalisib | Digoxin | 58.4 |
| 60 µM Amdizalisib | Digoxin | 75.9 |
| 80 µM Amdizalisib | Digoxin | 83.6 |
| BCRP inhibition | | |
| 1 µM Ko143 | E3S | 102 |
| 1 µM LY335979 | E3S | -11.8 |
| 0.1 µM Amdizalisib | E3S | 28.5 |
| 0.5 µM Amdizalisib | E3S | 34.2 |
| 2 µM Amdizalisib | E3S | 21.3 |
| 5 µM Amdizalisib | E3S | 24.2 |
| 10 µM Amdizalisib | E3S | 43.6 |
| 40 µM Amdizalisib | E3S | 52.9 |
| 60 µM Amdizalisib | E3S | 66.4 |
| 80 µM Amdizalisib | E3S | 52.0 |

1. **Inhibition of amdizalisib on activities of CYP isoforms**

**Table 6S** The percentage of enzyme activity remaining following incubation with amdizalisib.

| CYP isoforms | Duplicate | Amdizalisib concentration (μM) | | | | | | | | |
| --- | --- | --- | --- | --- | --- | --- | --- | --- | --- | --- |
|  |  | 0 | 0.08 | 0.4 | 2 | 5 | 10 | 25 |  | 50 |
| CYP3A4_ Testosterone | S1 | 95.6% | 101% | 86.1% | 89.9% | - | 83.5% | - |  | 86.7% |
|  | S2 | 104% | 99.4% | 90.5% | 94.3% | - | 84.8% | - |  | 69.0% |
| CYP2D6 | S1 | 100% | 103% | 103% | 104% | - | 98.0% | - |  | 80.0% |
|  | S2 | 99.7% | 103% | 107% | 98.0% | - | 98.6% | - |  | 84.5% |
| CYP2C9 | S1 | 97.6% | 98.3% | 94.2% | 82.6% | - | 53.7% | - |  | 17.2% |
|  | S2 | 102% | 99.7% | 95.6% | 82.6% | - | 52.8% | - |  | 16.0% |
| CYP2C19 | S1 | 95.8% | 97.1% | 105% | 102% | - | 96.6% | - |  | 68.6% |
|  | S2 | 104% | 111% | 99.5% | 98.0% | - | 88.1% | - |  | 65.9% |
| CYP3A4_ Midazolam | S1 | 96.7% | 93.8% | 106% | 103% | - | 99.4% | - |  | 89.1% |
|  | S2 | 103% | 105% | 103% | 100% | - | 101% | - |  | 86.3% |
| CYP2C8 | S1 | 104% | 110% | 105% | 98.8% | - | 67.3% | - |  | 38.0% |
|  | S2 | 95.7% | 105% | 109% | 104% | - | 76.5% | - |  | 39.6% |
| CYP1A2 | S1 | 95.6% | 104% | 102% | 94.1% | - | 97.6% | - |  | 99.1% |
|  | S2 | 104% | 96.1% | 99.1% | 95.9% | - | 90.1% | - |  | 99.6% |
| CYP2E1 | S1 | 101% | 99.7% | 99.0% | 101% | - | 105% | - |  | 118% |
|  | S2 | 99.0% | 97.6% | 96.9% | 95.5% | - | 97.6% | - |  | 113% |
| CYP2B6 | S1 | 100% | - | 100% | 96.2% | 97.4% | 97.7% | 89.5%87.6% |  | 79.7% |
|  | S2 | 100% | - | 98.9% | 92.1% | 95.1% | 96.6% | 87.6% |  | 77.1% |

Dash (-) indicates not determined; S1: sample 1; S2: sample 2.

1. **Evaluation of amdizalisib induction potential on CYP1A2, CYP2B6, CYP3A4, and CYP2C in human hepatocytes**

**Table 7S** CYP1A2, 2B6, and 3A4 activities and gene expression levels in human cryopreserved hepatocytes following incubation with amdizalisib and positive controls.

| Donor | CYP isoform | Compound | Conc. (µM) | Enzyme activity | | Gene expression | | | |
| --- | --- | --- | --- | --- | --- | --- | --- | --- | --- |
|  |  |  |  | Fold Induction | Percent of PC (%) | Fold Induction | Percent of PC (%) | E_max_ (fold) | EC_50_ (µM) |
| BXW | CYP1A2 | Omeprazole | 50 | 3.37 | - | 25.5 | - | - | - |
|  |  | Amdizalisib | 1 | 1.02 | 1.00 | 1.46 | 1.90 | 3.88 | 9.57 |
|  |  |  | 3 | 0.704 | -12.5 | 1.06 | 0.20 |  |  |
|  |  |  | 10 | 1.39 | 16.5 | 3.06 | 8.40 |  |  |
|  |  |  | 20 | 1.21 | 9.00 | 2.57 | 6.40 |  |  |
|  |  |  | 30 | 1.39 | 16.6 | 3.88 | 11.8 |  |  |
|  | CYP2B6 | Phenobarbital | 1000 | 7.19 | - | 9.45 | - | - | - |
|  |  | Amdizalisib | 1 | 1.34 | 5.50 | 1.80 | 9.50 | 5.42 | 5.73 |
|  |  |  | 3 | 0.871 | -2.10 | 1.15 | 1.70 |  |  |
|  |  |  | 10 | 2.17 | 19.0 | 6.14 | 60.8 |  |  |
|  |  |  | 20 | 1.10 | 1.70 | 2.89 | 22.4 |  |  |
|  |  |  | 30 | 1.12 | 2.00 | 4.69 | 43.7 |  |  |
|  | CYP3A4 | Rifampin | 1000 | 7.16 | - | 12.1 | - | - | - |
|  |  | Amdizalisib | 1 | 1.27 | 4.50 | 1.94 | 8.40 | 6.97 | 6.11 |
|  |  |  | 3 | 1.28 | 4.60 | 1.30 | 2.70 |  |  |
|  |  |  | 10 | 2.22 | 19.8 | 6.19 | 46.6 |  |  |
|  |  |  | 20 | 1.32 | 5.20 | 3.59 | 23.3 |  |  |
|  |  |  | 30 | 1.29 | 4.60 | 6.97 | 53.6 |  |  |
| XSM | CYP1A2 | Omeprazole | 50 | 4.95 | - | 15.6 | - | - | - |
|  |  | Amdizalisib | 1 | 1.09 | 2.40 | 1.27 | 1.80 | 2.17 | 5.50 |
|  |  |  | 3 | 1.15 | 3.90 | 1.06 | 0.40 |  |  |
|  |  |  | 10 | 1.30 | 7.60 | 2.49 | 10.3 |  |  |
|  |  |  | 20 | 1.59 | 14.9 | 2.06 | 7.30 |  |  |
|  |  |  | 30 | 1.29 | 7.30 | 1.95 | 6.60 |  |  |
|  | CYP2B6 | Phenobarbital | 1000 | 22.7 | - | 22.6 | - | - | - |
|  |  | Amdizalisib | 1 | 1.17 | 0.80 | 1.53 | 2.50 | 7.94 | 3.38 |
|  |  |  | 3 | 1.50 | 2.30 | 2.19 | 5.50 |  |  |
|  |  |  | 10 | 2.66 | 7.60 | 8.41 | 34.3 |  |  |
|  |  |  | 20 | 2.59 | 7.30 | 8.96 | 36.8 |  |  |
|  |  |  | 30 | 2.26 | 5.80 | 6.44 | 25.2 |  |  |
|  | CYP3A4 | Rifampin | 1000 | 2.98 | - | 159 | - | - | - |
|  |  | Amdizalisib | 1 | 1.01 | 0.50 | 2.59 | 1.00 | 35.7 | 9.91 |
|  |  |  | 3 | 1.07 | 3.60 | 3.52 | 1.60 |  |  |
|  |  |  | 10 | 1.39 | 19.8 | 19.7 | 11.9 |  |  |
|  |  |  | 20 | 1.57 | 28.9 | 34.9 | 21.4 |  |  |
|  |  |  | 30 | 1.35 | 17.7 | 35.5 | 21.9 |  |  |
| NFX | CYP1A2 | Omeprazole | 50 | 5.22 | - | 20.3 | - | - | - |
|  |  | Amdizalisib | 1 | 1.07 | 1.50 | 1.70 | 3.60 | 3.16 | 7.73 |
|  |  |  | 3 | 1.01 | 0.30 | 1.07 | 0.30 |  |  |
|  |  |  | 10 | 1.31 | 7.30 | 3.06 | 10.7 |  |  |
|  |  |  | 20 | 1.37 | 8.90 | 2.58 | 8.20 |  |  |
|  |  |  | 30 | 1.62 | 14.6 | 3.74 | 14.2 |  |  |
|  | CYP2B6 | Phenobarbital | 1000 | 3.66 | - | 9.27 | - | - | - |
|  |  | Amdizalisib | 1 | 1.07 | 2.60 | 1.45 | 5.40 | 3.39 | 4.48 |
|  |  |  | 3 | 1.08 | 3.00 | 1.19 | 2.30 |  |  |
|  |  |  | 10 | 1.08 | 3.10 | 3.45 | 29.6 |  |  |
|  |  |  | 20 | 0.704 | -11.1 | 2.50 | 18.1 |  |  |
|  |  |  | 30 | 0.647 | -13.3 | 4.22 | 39.0 |  |  |
|  | CYP3A4 | Rifampin | 1000 | 10.2 | - | 91.8 | - | - | - |
|  |  | Amdizalisib | 1 | 1.01 | 0.10 | 2.20 | 1.30 | >26.5 | >30.0 |
|  |  |  | 3 | 1.03 | 0.40 | 1.59 | 0.60 |  |  |
|  |  |  | 10 | 1.40 | 4.40 | 15.5 | 15.9 |  |  |
|  |  |  | 20 | 1.12 | 1.30 | 13.1 | 13.4 |  |  |
|  |  |  | 30 | 1.13 | 1.40 | 26.5 | 28.1 |  |  |

PC: Positive Control; E_max_, fold increase value at which there is maximum induction effect; EC_50_, concentration at which there is 50% of maximum induction effect.

**Table 8S** CYP2C8, 2C9, and 2C19 activities and gene expression levels in human cryopreserved hepatocytes following incubation with amdizalisib, negative and positive controls.

| Donor | CYP isoform | Compound | Conc.  (µM) | Enzyme activity | | | Gene expression | | |
| --- | --- | --- | --- | --- | --- | --- | --- | --- | --- |
|  |  |  |  | Fold Induction | Percent of PC (%) | Fold Induction | | Percent of PC (%) | |
| GKJ | CYP 2C8 | Flumazenil | 25 | 1.39 | 9.95 | | 0.92 | | -3.48 |
|  |  | Rifampin | 25 | 4.92 | - | | 3.23 | | - |
|  |  | Amdizalisib | 0.3 | 1.53 | 13.5 | | 1.05 | | 2.45 |
|  |  |  | 3 | 1.55 | 14.0 | | 1.32 | | 14.5 |
|  |  |  | 20 | 0.28 | -18.4 | | 0.92 | | -3.67 |
|  | CYP2C9 | Flumazenil | 25 | 1.14 | 10.2 | | 1.52 | | 19.7 |
|  |  | Rifampin | 25 | 2.37 | - | | 3.64 | | - |
|  |  | Amdizalisib | 0.3 | 1.08 | 5.84 | | 1.43 | | 16.1 |
|  |  |  | 3 | 1.41 | 29.9 | | 1.76 | | 28.8 |
|  |  |  | 20 | 1.50 | 36.5 | | 3.16 | | 81.6 |
|  | CYP2C19 | Flumazenil | 25 | 0.91 | -3.91 | | - | | - |
|  |  | Rifampin | 25 | 3.30 | - | | - | | - |
|  |  | Amdizalisib | 0.3 | 0.95 | -2.17 | | - | | - |
|  |  |  | 3 | 0.69 | -13.5 | | - | | - |
|  |  |  | 20 | 0.35 | -28.3 | | - | | - |
| ZEY | CYP 2C8 | Flumazenil | 25 | 1.05 | 0.998 | | 1.05 | | 0.98 |
|  |  | Rifampin | 25 | 6.01 | - | | 6.05 | | - |
|  |  | Amdizalisib | 0.3 | 0.91 | -1.80 | | 1.03 | | 0.67 |
|  |  |  | 3 | 0.34 | -13.2 | | 1.45 | | 9.01 |
|  |  |  | 20 | 0.19 | -16.2 | | 0.86 | | -2.83 |
|  | CYP2C9 | Flumazenil | 25 | 1.05 | 2.91 | | 1.53 | | 12.1 |
|  |  | Rifampin | 25 | 2.72 | - | | 5.35 | | - |
|  |  | Amdizalisib | 0.3 | 1.00 | 0.00 | | 1.23 | | 5.23 |
|  |  |  | 3 | 1.24 | 14.0 | | 1.87 | | 20.0 |
|  |  |  | 20 | 1.25 | 14.5 | | 3.37 | | 54.5 |
|  | CYP2C19 | Flumazenil | 25 | 0.87 | -3.72 | | - | | - |
|  |  | Rifampin | 25 | 4.49 | - | | - | | - |
|  |  | Amdizalisib | 0.3 | 1.09 | 2.58 | | - | | - |
|  |  |  | 3 | 0.87 | -3.72 | | - | | - |
|  |  |  | 20 | 0.42 | -16.6 | | - | | - |
| WKF | CYP 2C8 | Flumazenil | 25 | 1.18 | 3.75 | | 1.89 | | 15.4 |
|  |  | Rifampin | 25 | 5.80 | - | | 6.78 | | - |
|  |  | Amdizalisib | 0.3 | 1.25 | 5.21 | | 1.42 | | 7.24 |
|  |  |  | 3 | 1.52 | 10.8 | | 1.78 | | 13.5 |
|  |  |  | 20 | 0.07 | -19.4 | | 1.23 | | 3.97 |
|  | CYP2C9 | Flumazenil | 25 | 1.11 | 6.88 | | 1.83 | | 25.6 |
|  |  | Rifampin | 25 | 2.60 | - | | 4.24 | | - |
|  |  | Amdizalisib | 0.3 | 1.09 | 5.63 | | 1.41 | | 12.7 |
|  |  |  | 3 | 1.48 | 30.0 | | 1.58 | | 18.0 |
|  |  |  | 20 | 1.61 | 38.1 | | 2.91 | | 59.0 |
|  | CYP2C19 | Flumazenil | 25 | 1.04 | 1.83 | | - | | - |
|  |  | Rifampin | 25 | 3.19 | - | | - | | - |
|  |  | Amdizalisib | 0.3 | 1.14 | 6.39 | | - | | - |
|  |  |  | 3 | 1.04 | 1.83 | | - | | - |
|  |  |  | 20 | 0.58 | -19.2 | | - | | - |

PC: Positive Control

**Table 9S** The percentage of amdizalisib remaining after the last incubation in CYP1A2, 2B6, and 3A4 induction assay

| Donor Lot No. | Conc.(µM) | The percentage of amdizalisib remaining (%) | | |
| --- | --- | --- | --- | --- |
|  |  | 0 h | 5 h | 24 h |
| BXW | 1 | 100.0% | 101.7% | 51.3% |
|  | 3 | 100.0% | 99.1% | 71.1% |
|  | 10 | 100.0% | 109.4% | 44.8% |
|  | 20 | 100.0% | 116.6% | 53.3% |
|  | 30 | 100.0% | 105.3% | 82.6% |
| XSM | 1 | 100.0% | 95.7% | 50.0% |
|  | 3 | 100.0% | 97.6% | 70.8% |
|  | 10 | 100.0% | 115.1% | 87.9% |
|  | 20 | 100.0% | 117.7% | 97.9% |
|  | 30 | 100.0% | 106.4% | 95.3% |
| NFX | 1 | 100.0% | 81.4% | 36.0% |
|  | 3 | 100.0% | 89.5% | 62.3% |
|  | 10 | 100.0% | 105.8% | 86.8% |
|  | 20 | 100.0% | 106.9% | 102.7% |
|  | 30 | 100.0% | 104.3% | 96.4% |

The percentage of amdizalisib remaining% = (The peak area ratio of amdizalisib to internal standard at any incubation time) / (The peak area ratio of compound to internal standard at 0 h) × 100

**Table 10S** The measured amdizalisib concentrations at different time points after the last incubation in CYP2C induction assay

| Conc.(µM) | Donor Lot No. | Amdizalisib concentration (μM, Mean±SD) | | | |
| --- | --- | --- | --- | --- | --- |
|  |  | 0h | 1h | 4h | 24h |
| 0.3 | GKJ | 0.383 ± 0.003 | 0.400 ± 0.006 | 0.387 ± 0.012 | 0.313 ± 0.020 |
|  | ZEY | 0.377 ± 0.009 | 0.380 ± 0.006 | 0.367 ± 0.022 | 0.197 ± 0.007 |
|  | WKF | 0.380 ± 0.012 | 0.373 ± 0.009 | 0.360 ± 0.006 | 0.270 ± 0.006 |
| 3 | GKJ | 3.30 ± 0.24 | 3.38 ± 0.33 | 3.01 ± 0.07 | 2.97 ± 0.25 |
|  | ZEY | 3.69 ± 0.34 | 3.57 ± 0.26 | 3.44 ± 0.24 | 3.08 ± 0.06 |
|  | WKF | 4.02 ± 0.23 | 4.14 ± 0.30 | 3.81 ± 0.15 | 3.61 ± 0.29 |
| 20 | GKJ | 26.9 ± 1.9 | 23.6 ± 0.59 | 25.0 ± 1.8 | 22.2 ± 0.44 |
|  | ZEY | 28.6 ± 0.68 | 23.3 ± 0.27 | 21.2 ± 2.3 | 21.9 ± 0.27 |
|  | WKF | 31.4 ± 2.0 | 24.7 ± 2.23 | 27.7 ± 4.9 | 20.9 ± 0.57 |

1. **Inhibition of OATP-, OAT-, OCT-, and MATE-mediated transports by amdizalisib**

**Table 11S** Inhibitory effects of amdizalisib and positive inhibitors on OAT1-mediated 4- aminohippuric acid, OAT3-mediated estrone 3-sulfate, OCT2-mediated metformin, OATP-mediated β-estradiol 17-(β-D-glucuronide) and MATE- mediated tetraethylammonium uptakes.

| Compound and Concentration | Uptake rate (pmol/mg protein/min) | Percentage transported (%) | Percentage inhibition (%) |
| --- | --- | --- | --- |
| OAT1 | | | |
| Vehicle control | 506±80.3 | 100 | NA |
| 30 μM Probenecid | 41.8±3.63 | 0.00 | 100 |
| 0.3 μM Amdizalisib | 489±79.6 | 96.4 | 3.65 |
| 1 μM Amdizalisib | 467±34.8 | 91.6 | 8.44 |
| 3 μM Amdizalisib | 278±37.1 | 50.9 | 49.1 |
| 10 μM Amdizalisib | 196±6.18 | 33.3 | 66.7 |
| 30 μM Amdizalisib | 76.5±17.7 | 7.47 | 92.5 |
| 100 μM Amdizalisib | 8.38±1.24 | -7.21 | 107 |
| OAT3 | | | |
| Vehicle control | 104±4.92 | 100 | NA |
| 100 μM Probenecid | 19.5±2.26 | 0.00 | 100 |
| 0.3 μM Amdizalisib | 101±1.22 | 96.4 | 3.57 |
| 1 μM Amdizalisib | 106±2.62 | 103 | -2.52 |
| 3 μM Amdizalisib | 102±3.76 | 97.5 | 2.54 |
| 10 μM Amdizalisib | 105±1.14 | 101 | -0.883 |
| 30 μM Amdizalisib | 63.6±3.38 | 52.3 | 47.7 |
| 100 μM Amdizalisib | 42.5±2.15 | 27.3 | 72.7 |
| OCT2 | | | |
| Vehicle control | 781±75.8 | 100 | NA |
| 300 μM Verapamil | 64.9±21.5 | 0.00 | 100 |
| 0.3 μM Amdizalisib | 772±136 | 98.7 | 1.28 |
| 1 μM Amdizalisib | 733±47.7 | 93.3 | 6.72 |
| 3 μM Amdizalisib | 746±13.4 | 95.1 | 4.92 |
| 10 μM Amdizalisib | 728±32.8 | 92.7 | 7.34 |
| 30 μM Amdizalisib | 839±49.9 | 108 | -8.07 |
| 100 μM Amdizalisib | 429±46.2 | 50.8 | 49.2 |
| OATP1B1 | | | |
| Vehicle control | 52.8±1.99 | 100 | NA |
| 10 μM Rifampicin | 3.03±0.536 | 0.00 | 100 |
| 0.3 μM Amdizalisib | 59.0±1.42 | 113 | -12.5 |
| 1 μM Amdizalisib | 55.1±2.95 | 105 | -4.64 |
| 3 μM Amdizalisib | 47.2±3.13 | 86.5 | 13.5 |
| 10 μM Amdizalisib | 19.3±4.10 | 32.7 | 67.3 |
| 30 μM Amdizalisib | 7.03±1.12 | 8.04 | 92.0 |
| 100 μM Amdizalisib | 1.46±0.141 | -3.17 | 103 |
| OATP1B3 | | | |
| Vehicle control | 65.8±5.80 | 100 | NA |
| 10 μM Rifampicin | 4.46±0.829 | 0.00 | 100 |
| 0.3 μM Amdizalisib | 62.4±5.58 | 94.5 | 5.47 |
| 1 μM Amdizalisib | 59.1±6.36 | 89.2 | 10.8 |
| 3 μM Amdizalisib | 46.2±10.1 | 68.1 | 31.9 |
| 10 μM Amdizalisib | 22.4±1.00 | 29.3 | 70.7 |
| 30 μM Amdizalisib | 6.76±0.372 | 3.82 | 96.2 |
| 100 μM Amdizalisib | 1.99±0.315 | -4.02 | 104 |
| MATE1 | | | |
| Vehicle control | 249±9.98 | 100 | NA |
| 1 μM Pyrimethamine | 12.5±2.43 | 0.00 | 100 |
| 0.3 μM Amdizalisib | 244±23.4 | 98.0 | 2.01 |
| 1 μM Amdizalisib | 216±1.37 | 86.1 | 13.9 |
| 3 μM Amdizalisib | 186±12.4 | 73.2 | 26.8 |
| 10 μM Amdizalisib | 129±1.08 | 49.2 | 50.8 |
| 30 μM Amdizalisib | 126±22.8 | 47.8 | 52.2 |
| 100 μM Amdizalisib | 68.4±2.28 | 23.6 | 76.4 |
| MATE2-K | | | |
| Vehicle control | 142±6.71 | 100 | NA |
| 1 μM Pyrimethamine | 7.55±1.58 | 0.00 | 100 |
| 0.3 μM Amdizalisib | 125±3.49 | 87.1 | 12.9 |
| 1 μM Amdizalisib | 129±7.96 | 89.9 | 10.1 |
| 3 μM Amdizalisib | 127±5.50 | 89.0 | 11.0 |
| 10 μM Amdizalisib | 128±4.89 | 89.6 | 10.4 |
| 30 μM Amdizalisib | 73.9±6.49 | 49.3 | 50.7 |
| 100 μM Amdizalisib | 47.4±3.61 | 29.6 | 70.4 |

NA: not applicable

_
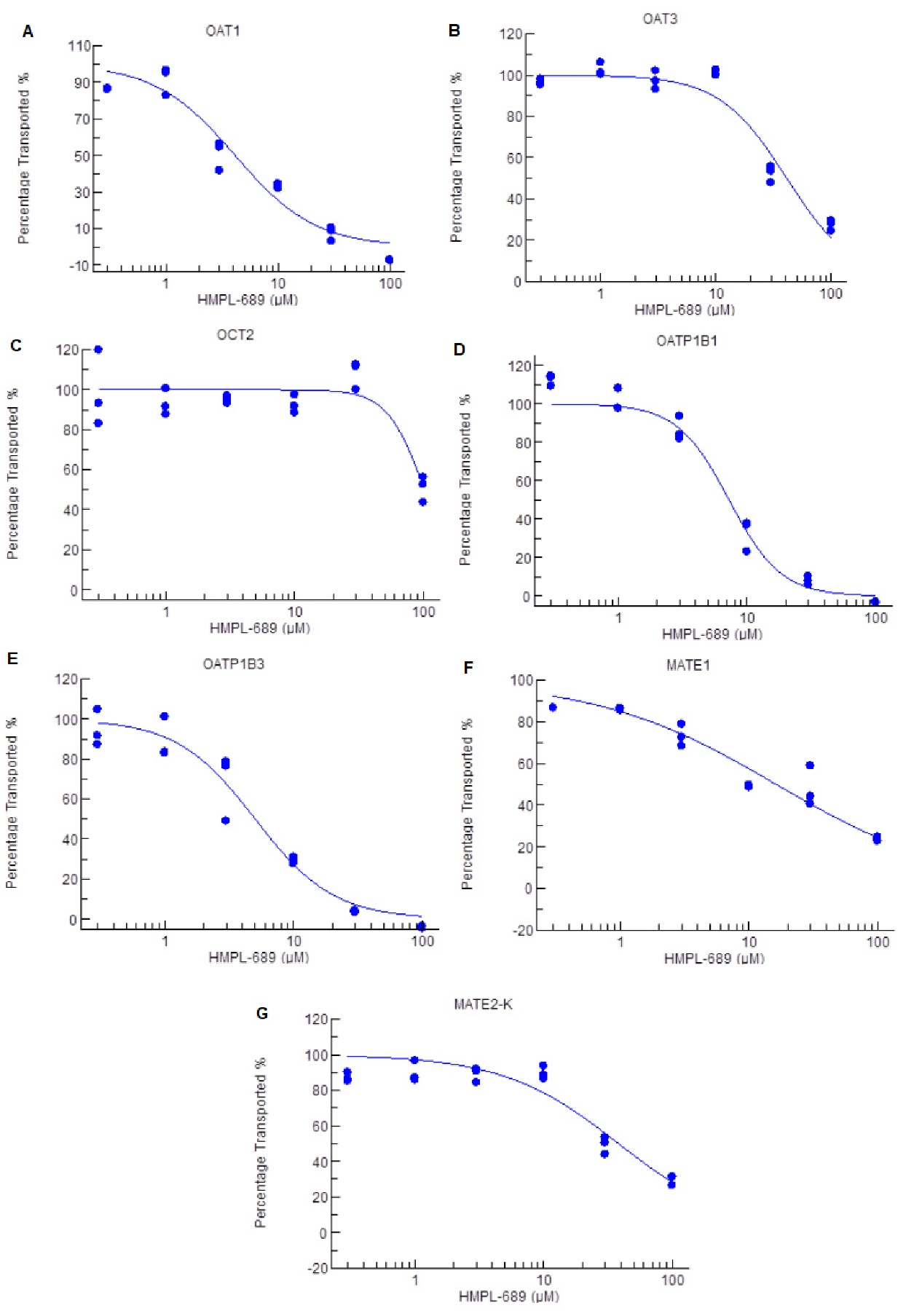
_

**Figure 1S** The concentration-dependent inhibition of amdizalisib on the substrate uptakes mediated by human (**A**) OAT1, (**B**) OAT3, (**C**) OCT2, (**D**) OATP1B1, (**E**) OATP1B3, (**F**) MATE1 and (**G**) MATE2-K over the concentration range of 0.3-100 μM.
